# Discriminatory plasma biomarkers predict specific clinical phenotypes of necrotizing soft-tissue infections

**DOI:** 10.1172/JCI149523

**Published:** 2021-07-15

**Authors:** Laura M. Palma Medina, Eivind Rath, Sanjeevan Jahagirdar, Trond Bruun, Martin B. Madsen, Kristoffer Strålin, Christian Unge, Marco Bo Hansen, Per Arnell, Michael Nekludov, Ole Hyldegaard, Magda Lourda, Vitor A.P. Martins dos Santos, Edoardo Saccenti, Steinar Skrede, Mattias Svensson, Anna Norrby-Teglund

**Affiliations:** 1 Center for Infectious Medicine, Department of Medicine Huddinge, Karolinska Institute, Stockholm, Sweden.; 2 Department of Medicine, Division for Infectious Diseases, Haukeland University Hospital, Bergen, Norway.; 3 Laboratory of Systems and Synthetic Biology, Wageningen University and Research, Wageningen, Netherlands.; 4 Department of Clinical Science, University of Bergen, Bergen, Norway.; 5 Department of Intensive Care, Copenhagen University Hospital, Rigshospitalet, Copenhagen, Denmark.; 6 Department of Medicine Huddinge, Karolinska Institute, Stockholm, Sweden.; 7 Department of Infectious Diseases and; 8 Functional Area of Emergency Medicine, Karolinska University Hospital, Stockholm, Sweden.; 9 Department of Anaesthesia, Centre of Head and Orthopaedics, Copenhagen University Hospital, Rigshospitalet, Copenhagen, Denmark.; 10 Department of Anaesthesia and Intensive Care, Sahlgrenska University Hospital, Gothenburg, Sweden.; 11 Department of Anaesthesia, Surgical Services and Intensive Care, Karolinska Institute, Karolinska University Hospital, Stockholm, Sweden.; 12 Childhood Cancer Research Unit, Department of Women’s and Children’s Health, Karolinska Institutet, Stockholm, Sweden.; 13 LifeGlimmer GmbH, Berlin, Germany.

**Keywords:** Immunology, Infectious disease, Bacterial infections, Diagnostics

## Abstract

**BACKGROUND:**

Necrotizing soft-tissue infections (NSTIs) are rapidly progressing infections frequently complicated by septic shock and associated with high mortality. Early diagnosis is critical for patient outcome, but challenging due to vague initial symptoms. Here, we identified predictive biomarkers for NSTI clinical phenotypes and outcomes using a prospective multicenter NSTI patient cohort.

**METHODS:**

Luminex multiplex assays were used to assess 36 soluble factors in plasma from NSTI patients with positive microbiological cultures (*n* = 251 and *n* = 60 in the discovery and validation cohorts, respectively). Control groups for comparative analyses included surgical controls (*n* = 20), non-NSTI controls (i.e., suspected NSTI with no necrosis detected upon exploratory surgery, *n* = 20), and sepsis patients (*n* = 24).

**RESULTS:**

Thrombomodulin was identified as a unique biomarker for detection of NSTI (AUC, 0.95). A distinct profile discriminating mono- (type II) versus polymicrobial (type I) NSTI types was identified based on differential expression of IL-2, IL-10, IL-22, CXCL10, Fas-ligand, and MMP9 (AUC >0.7). While each NSTI type displayed a distinct array of biomarkers predicting septic shock, granulocyte CSF (G-CSF), S100A8, and IL-6 were shared by both types (AUC >0.78). Finally, differential connectivity analysis revealed distinctive networks associated with specific clinical phenotypes.

**CONCLUSIONS:**

This study identifies predictive biomarkers for NSTI clinical phenotypes of potential value for diagnostic, prognostic, and therapeutic approaches in NSTIs.

**TRIAL REGISTRATION:**

ClinicalTrials.gov NCT01790698.

**FUNDING:**

Center for Innovative Medicine (CIMED); Region Stockholm; Swedish Research Council; European Union; Vinnova; Innovation Fund Denmark; Research Council of Norway; Netherlands Organisation for Health Research and Development; DLR Federal Ministry of Education and Research; and Swedish Children’s Cancer Foundation.

## Graphical abstract

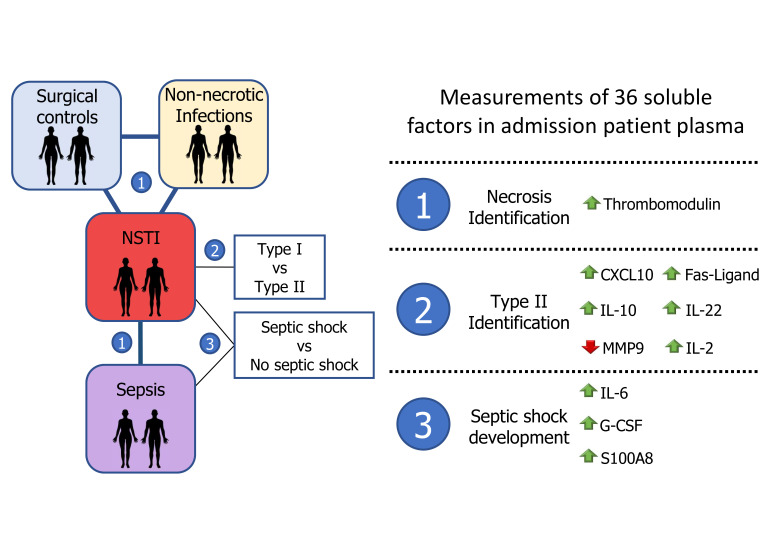

## Introduction

Necrotizing soft-tissue infections (NSTIs) are characterized by extensive damage in any layer of the soft-tissue compartment (1, 2). These infections are infrequent, but are associated with a significant health burden due to high mortality and risk of severe long-term disability as a consequence of extensive tissue loss or amputations (3–5). The progression of the disease is rapid, and early identification is therefore pivotal for improving the prognosis of affected patients. Currently, the initial diagnosis of NSTI is challenging due to the often vague symptoms during early stages, a heterogeneous patient group, and lack of specific diagnostic tools (6), which lead to misdiagnoses of NSTI in many cases (7). Still, doctors are advised that in case of NSTI suspicion, patients should be referred to surgical evaluation immediately (8). Previous efforts to improve the diagnosis of NSTIs led to the proposal of the Laboratory Risk Indicator for Necrotizing Fasciitis (LRINEC) (9). However, its utility has been disproven due to low sensitivity (5, 8, 10, 11). Therefore, there is still a need for early diagnostic tools facilitating the swift detection of NSTI cases and thereby enabling prompt and adequate treatment (12, 13).

NSTIs are often classified based on etiology in which 4 types of infections are distinguished; however, the majority of cases consist of types I and II (14, 15). Type I NSTIs are caused by polymicrobial communities working synergistically. This type of infection is the most common type of NSTI, affecting primarily elderly patients and patients with underlying conditions (2). These pathogenic communities include anaerobic and often also aerobic bacteria, including *Escherichia coli* or *Pseudomonas* sp., among others (15, 16). In contrast, type II infections are caused by a single bacteria l species, most predominantly by β-hemolytic streptococci, of which *Streptococcus pyogenes* (group A Streptococcus [GAS]) is the most common, followed by *Streptococcus* *dysgalactiae* (17). This type of NSTI occurs primarily in the extremities of patients that tend to be younger and more often without underlying conditions (5, 14, 17). Moreover, GAS NSTI cases are often complicated by toxic shock syndrome (5, 17–20). The diversity of microbiological etiologies of these severe infections should translate into different underlying pathogenic mechanisms. In fact, NSTI type–specific host-pathogen interactions were identified by Thänert et al. (21) using dual RNA-Seq analyses of tissue biopsies from NSTI patients. This highlighted the possibility of developing diagnostic tools that can contribute to identifying NSTI clinical phenotypes and predicting outcome, thereby supporting therapeutic strategies that target specific pathogenic mechanisms.

Although these infections are localized in the deep soft tissue, systemic complications are frequently seen and inflammatory mediators have been measured in circulation (22). This shows the potential of a diagnostic tool assessing biomarkers in blood, which is advantageous in terms of sampling and options for rapid tests (6, 23). In the present study, we explored 36 plasma molecules as potential biomarkers for detection and characterization of clinical phenotypes of NSTI using the NSTI patient cohort collected through the prospective multicenter INFECT study (24), in which distinct clinical phenotypes involving different comorbidities, localization, and microbiological etiology were identified (5, 17). We used univariate, multivariate, machine learning, and differential connectivity analyses to identify predictive biomarker sets linked to unique NSTI clinical phenotypes.

## Results

### Study subjects for the discovery cohort.

**Figure 1:**
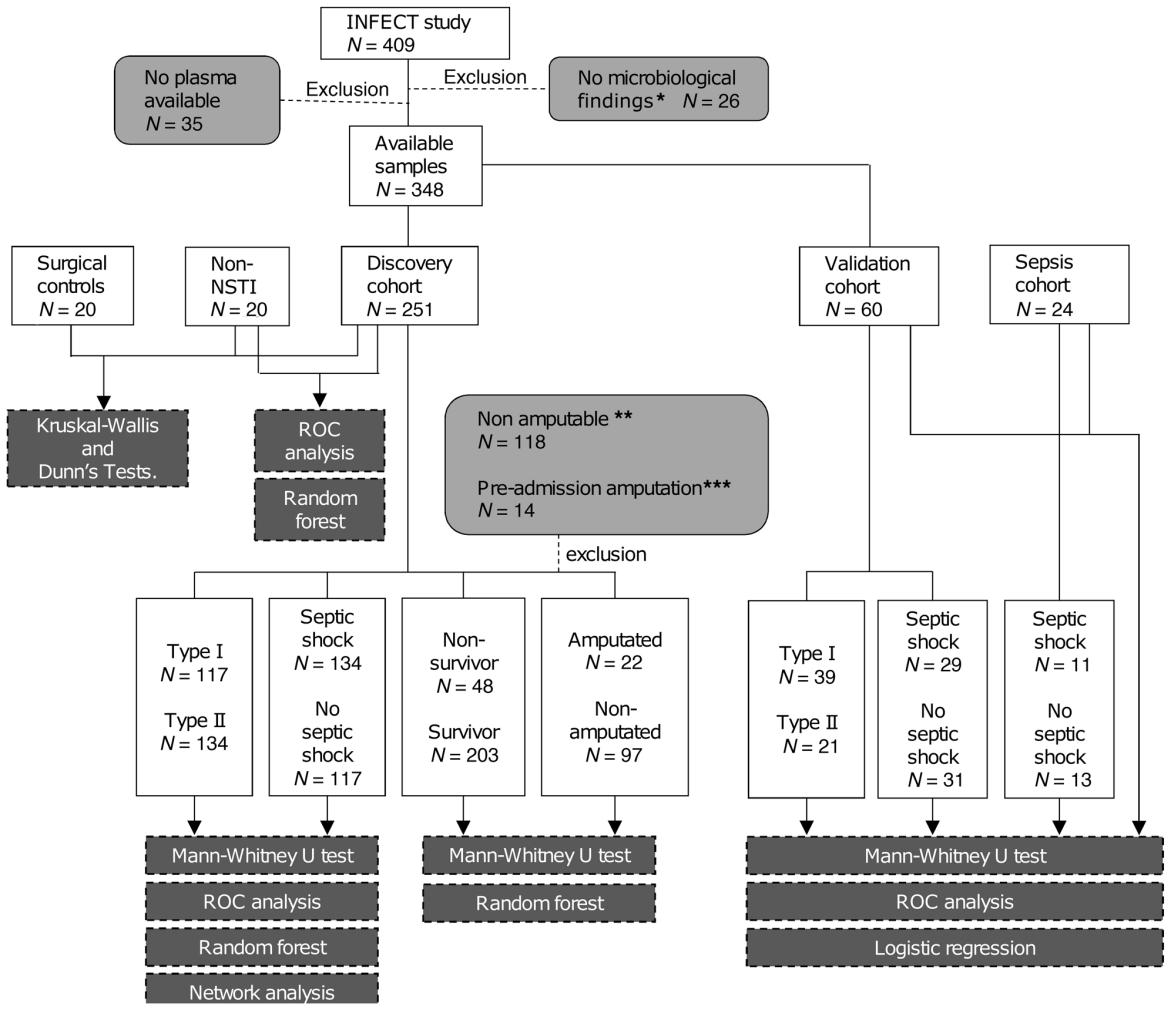
Flow chart of the study pipeline. The samples included in each test are displayed inside solid line boxes, light gray boxes show the reasons for exclusion at different stages of the study, and dark gray boxes indicate the specific test applied to the different set of samples. *Plasma samples from the INFECT cohort were excluded from the study if there was no positive microbiological culture in blood or tissue. Samples from patients with NSTI in nonamputable sites (i.e., neck, abdomen, and thorax) (**) or who had undergone amputation before admission (***) were not included for the prediction model for amputation.

Study subjects were selected from the INFECT patient cohort (5, 24). A key aspect of this study was to ensure that the microbiological etiology was considered, as this influences the clinical phenotypes and the pathogenic mechanisms. For this purpose, only patients with positive microbiological culture in blood or tissue and with plasma collected at the time of enrollment were included in the analysis. Out of the 348 patients in the INFECT cohort with microbiological results and available plasma samples, 251 patients were selected for the discovery cohort (Figure 1). These included 117 type I (47%) and 134 type II (53%) NSTI cases, thus obtaining an etiology distribution that was representative of the original INFECT cohort (5). Plasma samples from 2 control groups were also included in the analyses: 20 patients with suspected NSTI in whom no necrotic tissue was found upon explorative surgery (non-NSTI controls) and an additional control group of 20 patients who had surgical procedures not related to infection (surgical controls). The latter cohort was matched with the NSTI patients for age and sex. The associated clinical and microbiological data of the patients and controls are shown in Table 1. The distribution of age, sex, and simplified acute physiology score II (SAPS II) (25) was similar among all NSTI patients regardless of the type of infection.

### Biomarkers discriminating NSTI from non-NSTI controls.

**Figure 2:**
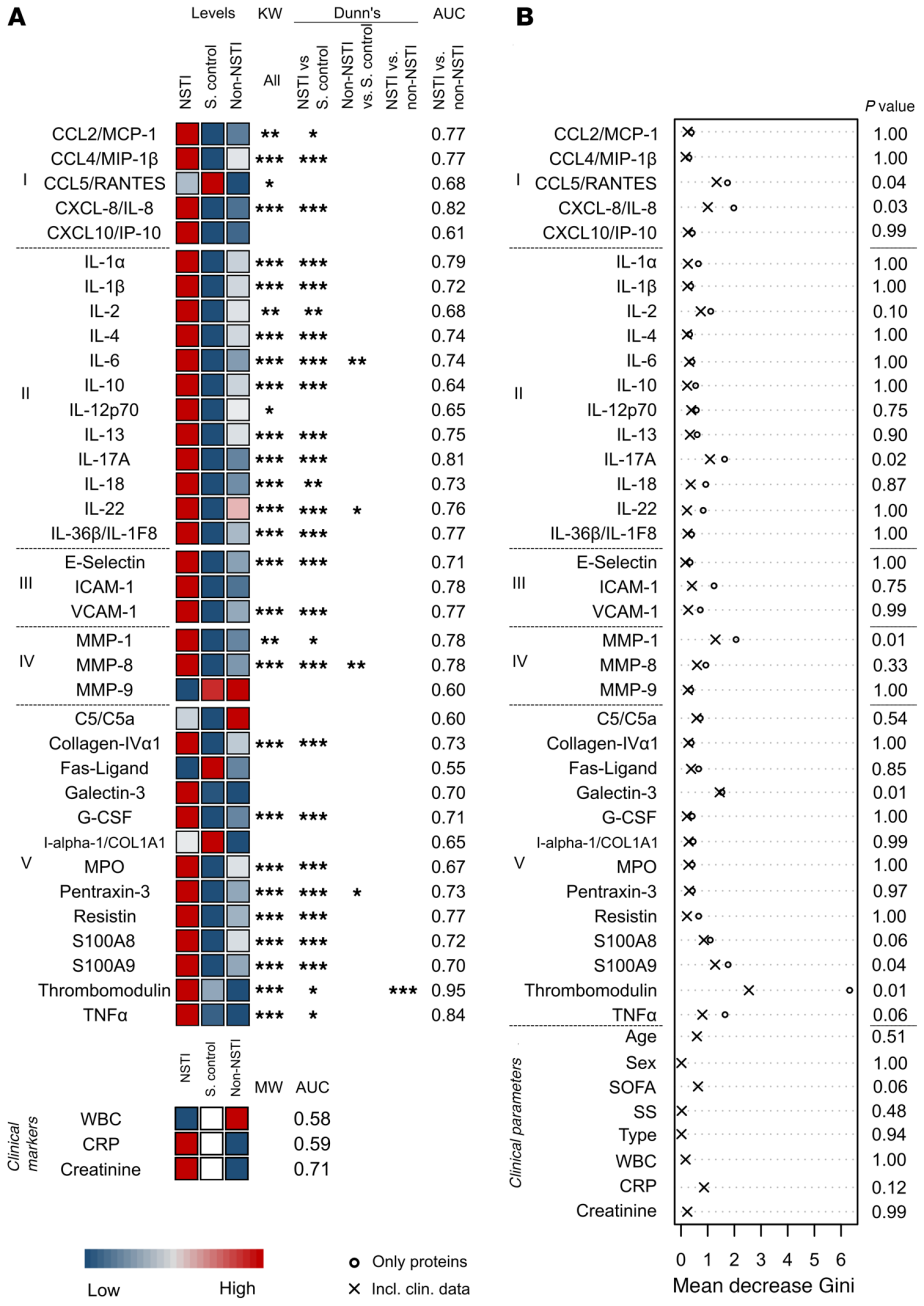
Thrombomodulin is a plasma protein with biomarker potential for discrimination of NSTI and non-NSTI. Concentrations of the soluble factors in plasma were compared among NSTI patients (*n* = 251), surgical controls (S. control; *n* = 20), and non-NSTI controls (*n* = 20). (**A**) The median protein levels in each cohort are depicted in the heatmap. All individual values are shown in Supplemental Figure 1. The measured proteins are divided by categories: I, chemokines; II, interleukins; III, soluble adhesion molecules; IV, matrix metalloproteases; and V, others. Significant differences between the measured concentrations were tested using Kruskal-Wallis (KW) test followed by Dunn’s post hoc test or Mann-Whitney *U* test (MW). Asterisks indicate the *q* cutoff obtained in at least 95% of the iterations. *q = 0.05; **q = 0.01; ***q = 0.005. The AUCs from the ROC analyses are given as the mean values of the iterations. The confidence intervals, specificities, and sensitivities of this test are included in Supplemental Table 1. (**B**) The RF result for discriminating NSTI versus non-NSTI is presented as the mean decrease Gini for each variable. The displayed *P* values are the result of the model including clinical data (Supplemental Table 1). SS, septic shock; type, microbiological classification of NSTI.

To pinpoint relevant markers for early detection of NSTI, a customized Luminex multiplex assay including 36 soluble factors involved in inflammatory responses and tissue remodeling was designed. The biomarker profiles in plasma samples from patients were compared with those of controls, and as expected, the highest concentrations of the markers were typically measured in plasma from NSTI patients, followed by the non-NSTI controls, and finally, the noninfected surgical controls (Figure 2A and Supplemental Figure 1; supplemental material available online with this article; https://doi.org/10.1172/JCI149523DS1). The use of a stringent statistical analysis allowed the identification of the most robust biomarkers discriminating between the groups. The results revealed that most analytes’ concentrations were significantly higher in NSTI samples than in the noninfected surgical controls (*q* < 0.05), whereas only 4 markers, i.e., IL-6, IL-22, MMP-8, and pentraxin-3, were significantly higher in non-NSTI samples compared with those from the surgical controls (*q* < 0.05). Most relevant from a clinical perspective, comparison between NSTI and non-NSTI cases revealed that only thrombomodulin differed significantly between these groups (*q* < 0.0005) (Figure 2A). The robustness of this protein as a potential biomarker was corroborated by receiver operating characteristic (ROC) analysis with an AUC of 0.95 (specificity, 0.89; sensitivity, 0.92, at a concentration threshold of 7567 pg/ml), outperforming selected clinical markers (Supplemental Figure 2, A and B, and Supplemental Table 1). Next, multivariate analysis using random forest (RF) modeling, including the whole biomarker set and key clinical parameters (i.e., age, sex, sequential organ failure assessment [SOFA] score (26), septic shock, NSTI type, WBC, C-reactive protein [CRP] and creatinine), was used to identify biomarkers predictive of NSTI. The analyses identified a set of 5 biomarkers, i.e., IL-17A, galectin-3, S100A8, S100A9, and thrombomodulin, that differentiated between NSTI and non-NSTI cases (Figure 2B and Supplemental Table 1). Notably, thrombomodulin was the most robust predictive marker even in the multivariate model. Furthermore, comparison of NSTI patients divided based on early (severe pain, in need of opioids), intermediate (skin bullae or skin bruising), and late (skin purple/black discoloration, skin anesthesia, palpable gas [crepitus] or gas visualized on radiology) signs of NSTI revealed that even patients with only early signs had significantly higher levels of thrombomodulin than non-NSTI controls (Supplemental Figure 2C). Moreover, these levels further increased in patients with intermediate or late signs of NSTI.

### Biomarkers discriminating between type I and type II NSTIs.

**Figure 3:**
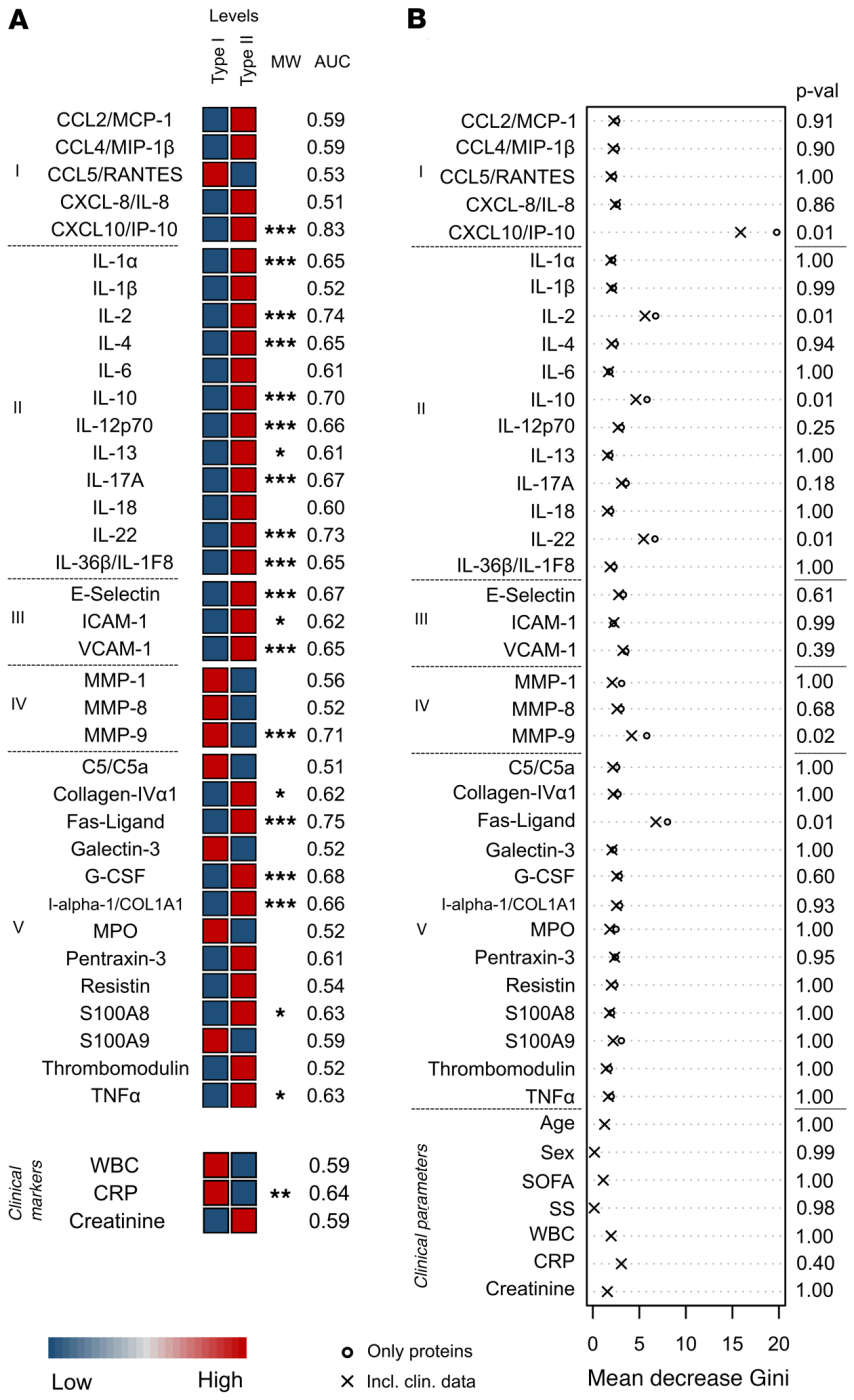
Biomarker panel for discrimination of type I and type II. Levels of the soluble factors in plasma were compared between type I (*n* = 117) and type II (*n* = 134) patients within the NSTI discovery cohort (Table 1). (**A**) Heatmap depicting the median protein levels in each NSTI type. The measured proteins are divided by categories: I, chemokines; II, interleukins; III, soluble adhesion molecules; IV, matrix metalloproteases; and V, others. Significant differences between the measured concentrations were tested using Mann-Whitney *U* test. Asterisks indicate the *q* cutoff obtained in at least 95% of the results. *q = 0.05; **q = 0.01; ***q = 0.005. AUCs from the ROC analyses are shown as the mean values of the iterations. The confidence intervals, specificities, and sensitivities of this test are shown in Supplemental Table 2. (**B**) The RF result is shown as the mean decrease Gini for each variable. The displayed *P* values are the result of the model including clinical data (Supplemental Table 2).

Comparison of the inflammatory response in the 2 types of NSTIs revealed distinct profiles (Figure 3A). Type II NSTI patients tended to have higher concentrations of the inflammatory markers, while type I had higher levels of the MMPs. Among the 20 biomarkers with significant differences between the NSTI types, only 6 (i.e., CXCL10/IP-10, IL-2, IL-10, IL-22, MMP-9, Fas-ligand) had an AUC greater than 0.7 (Supplemental Table 2), suggesting discriminatory potential. The same set of biomarkers was identified as predictive when the multivariate RF analysis was applied (Figure 3B and Supplemental Table 2). Notably, CXCL10/IP-10 was the biomarker with the highest AUC (0.83; Supplemental Table 2) in the univariate analysis as well as the highest mean decreased Gini in the RF model with a significant *P* value (<0.05).

### In vitro testing of biomarkers for type differentiation.

**Figure 4:**
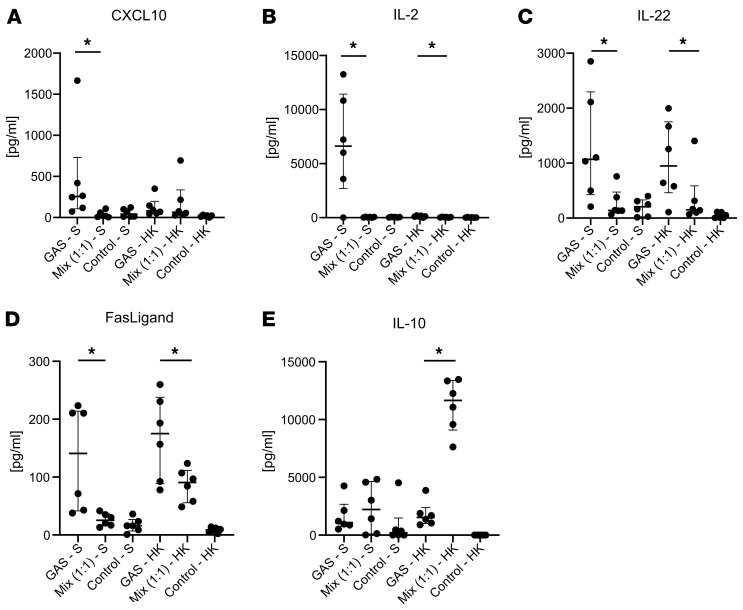
Differential production of selected proteins by PBMCs after in vitro stimulation with GAS compared with *B.* ***fragilis*plus*E. coli*(mix)**. Stimulations were conducted in 6 repeat experiments using PBMCs from different donors stimulated with bacterial supernatant (S) or HK bacteria. (**A**–**E**) Scatter plots of each measured analyte in the supernatant after 24 hours of stimulation. The graphs display the individual values and the median with interquartile range. *P < 0.05, Wilcoxon’s matched pairs signed rank test.

To further validate the type-specific biomarker panel, we tested to determine whether representative type I and type II NSTI clinical bacterial strains trigger differential inflammatory responses in line with those noted in patient plasma. For this purpose, human peripheral blood mononuclear cells (PBMCs) from healthy donors were stimulated with clinical NSTI bacterial strains. One GAS strain (*emm*1 type; strain 2006) was selected for the type II infection, while a mix of equal parts of *Bacteroides* *fragilis* and *E*. *coli* isolated from the same NSTI patient (patient 4011) was used to model a type I NSTI. These species were selected, as they were most frequently cultured in type I patients in the INFECT cohort (5). The bacterial stimuli included both supernatants containing extracellular factors as well as heat-killed (HK) bacteria for surface-attached factors. Although part of the biomarker panel, MMP-9 was excluded from the in vitro experiment, as PBMCs are not a major cellular source of this factor (27). In line with the different plasma concentrations, elevated levels of IL-2, IL-22, CXCL10, and Fas-ligand were found in type II– versus type I–stimulated cultures (Figure 4, A–D). In contrast, IL-10 was higher in cells stimulated with HK type I isolates versus the type II GAS isolate (Figure 4E); therefore, IL-10 showed the opposite result of that seen with the patient data.

### Biomarkers discriminating between NSTIs with or without septic shock.

**Figure 5:**
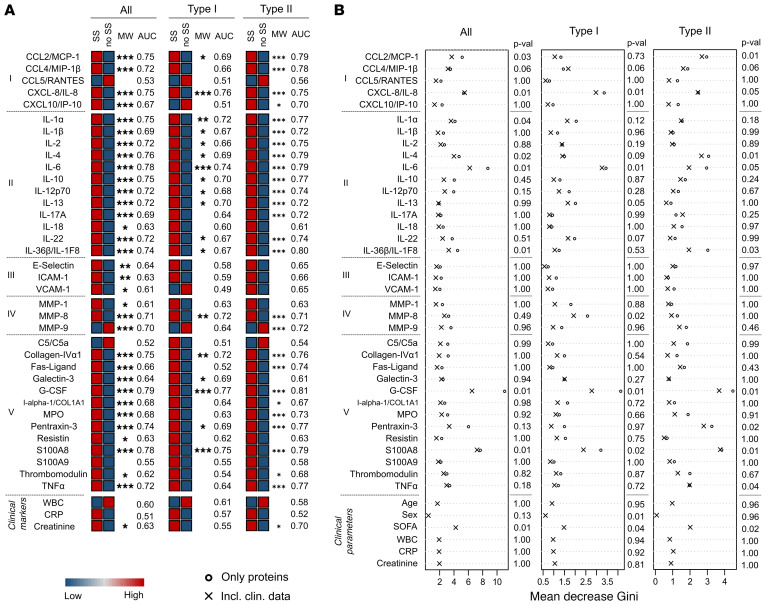
Biomarker signatures associated with septic shock differ depending on etiology of NSTI. Levels of the soluble factors in plasma were compared between patients with (*n* = 134) and without septic shock (*n* = 117) at admission within the NSTI discovery cohort (Table 1). (**A**) Heatmaps of the median protein concentrations in each phenotype. The measured proteins are divided by categories: I, chemokines; II, interleukins; III, soluble adhesion molecules; IV, matrix metalloproteases; and V, others. Significant differences between the measured concentrations were tested using Mann-Whitney *U* test. Asterisks indicate the *q* value cutoff obtained in at least 95% of the results. *q = 0.05; **q = 0.01; ***q = 0.005. The results from the ROC analysis are shown as the mean AUC values from the iterations. The confidence intervals, specificities, and sensitivities of this test are shown in Supplemental Table 3. (**B**) RF results are shown as the mean decrease Gini for each variable. Displayed *P* values are the results of the models including clinical data (Supplemental Table 4).

Biomarkers associated with severe outcome of NSTIs, such as septic shock, amputation, or death, were also explored within the NSTI cohort. The analyses revealed no significant changes linked to amputation or fatal outcome (Supplemental Figure 3), whereas septic shock was linked to marked differences in inflammatory profile (Figure 5A). Most analytes were significantly higher in plasma of patients with septic shock (*q* < 0.05). However, this was particularly evident in type II cases with or without septic shock, while in type I cases, fewer markers were significantly different (Figure 5A). Notably, 3 plasma proteins (i.e., IL-6, granulocyte CSF [G-CSF], and S100A8) were identified as potential biomarkers for septic shock regardless of NSTI type (Figure 5B and Supplemental Tables 3 and 4).

### Validation of identified biomarker panels in additional patient cohorts.

To test the veracity of the biomarker panels for identification of NSTIs and associated clinical phenotypes (microbiological etiology and septic shock), a validation cohort was analyzed. This cohort comprised 60 additional NSTI patients from the INFECT study (Figure 1). To further test the septic shock biomarker panel, 24 patients with sepsis (42% septic shock; no NSTI) of varying etiology were included (Supplemental Table 5). Due to the exclusive nature of the non-NSTI control group, it was not possible to retrieve similar samples for validation, and instead the sepsis cohort was also used as a comparative cohort to test the predictive value of the NST-associated biomarker thrombomodulin. The discovery and validation NSTI cohorts were well matched with respect to age, sex, and severity of infection. However, the microbiological etiology differed between cohorts, with GAS being significantly more prevalent in the discovery cohort (Supplemental Tables 5 and 6).

**Figure 6:**
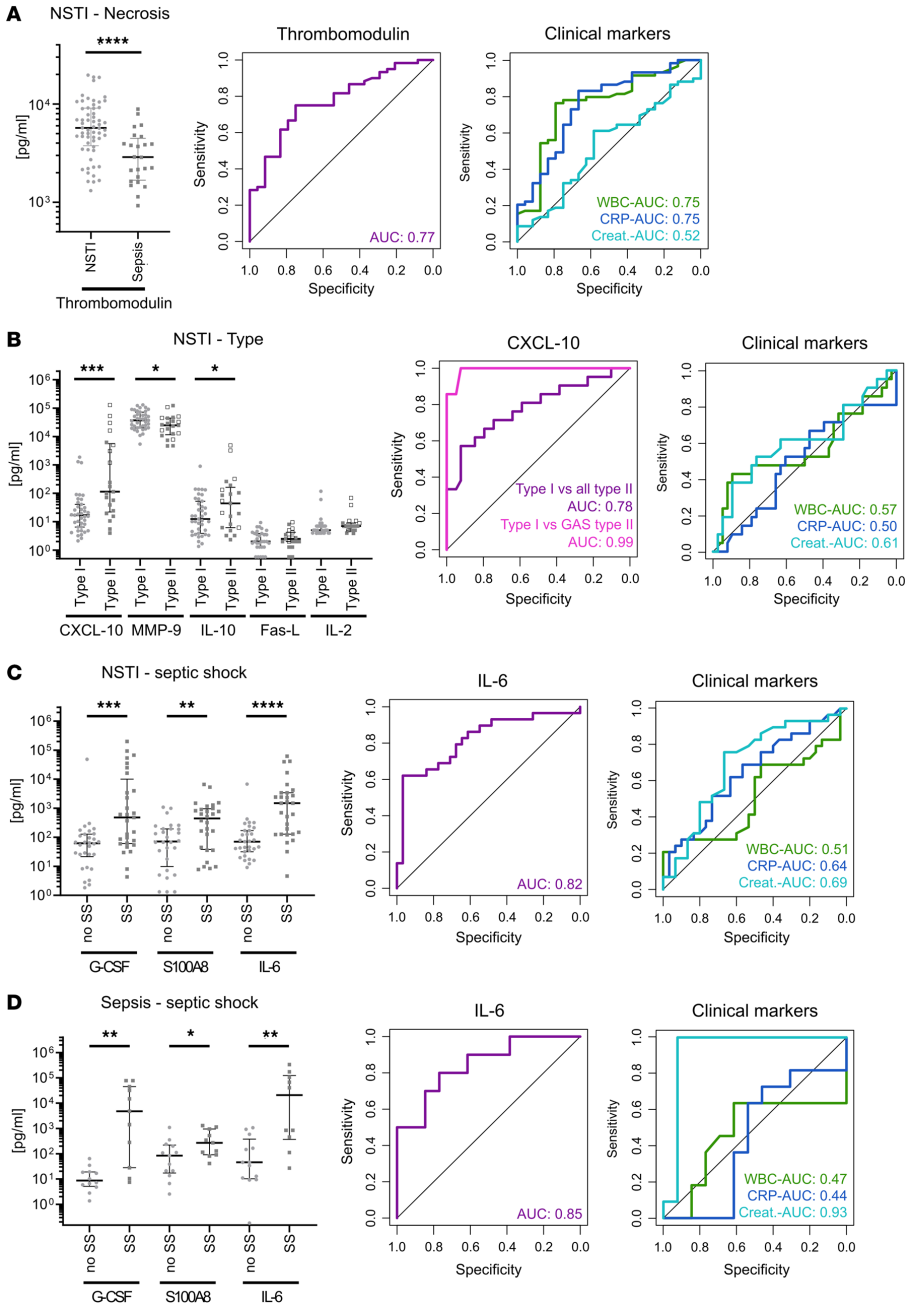
Predictive power of plasma biomarkers assessed in the validation cohort. Selected biomarkers were tested for their potential to detect (**A**) NSTI (necrosis), (**B**) NSTI type, and (**C** and **D**) septic shock. Scatter plots display the individual values and the median with interquartile range. The discovery cohort consist of 60 NSTI patients, of which 39 were type I and 29 developed septic shock (Supplemental Table 5). In panel **B**, empty squares indicate type II NSTI caused by GAS (*n* = 7). The control group of 24 sepsis patients included 11 patients with septic shock. *P < 0.05; **P < 0.01; ***P < 0.001; ****P < 0.0001; Mann-Whitney *U* test. ROC plots display results of the indicated biomarkers or clinical markers.

All selected biomarkers, including thrombomodulin, CXCL10/IP-10, IL-10, MMP-9, G-CSF, S1000A8, IL-6, IL-2, Fas-ligand, and IL-22, were measured in the validation cohort. However, the results of IL-22 were excluded due to a high number of left-censored data. The measured concentrations of the biomarkers were in the same order of magnitude as in the discovery cohort (Figure 6, A–D). The suggested biomarker for necrosis, thrombomodulin, showed a high discriminatory power for NSTIs even when compared with the heterogeneous sepsis patient group (Figure 6A and Supplemental Table 7).

Among the biomarkers discriminating between type I and type II NSTIs, CXCL10/IP10, MMP-9, IL-10, Fas-ligand, and IL-2, only the first 3 showed significant differences between type I and type II, whereas Fas-ligand and IL-2 did not (Figure 6B). The best performance was noted with CXCL10/IP-10 (AUC, 0.78; Supplemental Table 7). Since the prevalence of GAS in type II NSTI cases was significantly lower in the validation versus the discovery cohort (38% and 65%, respectively; Supplemental Tables 5 and 6), we tested the biomarker panel for comparison of type I versus only GAS type II infections. Notably, the predictive power of CXCL10 reached an impressive AUC of 0.99 (Figure 6B and Supplemental Table 7).

The biomarker panel associated with septic shock (i.e., IL-6, G-CSF, and S100A8) in NSTI patients was tested, and the results corroborated the previous findings based on the discovery cohort (Figure 6C). The value of these 3 biomarkers was also tested in the sepsis cohort, revealing a similar discriminatory power (Figure 6D and Supplemental Table 7). Among these biomarkers, IL-6 showed the best performance, with AUCs of 0.82 and 0.85 in the NSTI and sepsis cohorts, respectively. In line with creatinine being a definition marker for acute kidney injury and sepsis-associated organ failure, creatinine in the sepsis cohort showed an AUC of 0.93 (Figure 6D). Finally, multivariate logistic regression revealed that all biomarkers retained their discriminatory power for the specific NSTI clinical phenotypes even when sex, age, and SOFA scores were considered (Supplemental Table 8).

### Network analysis.

**Figure 7:**
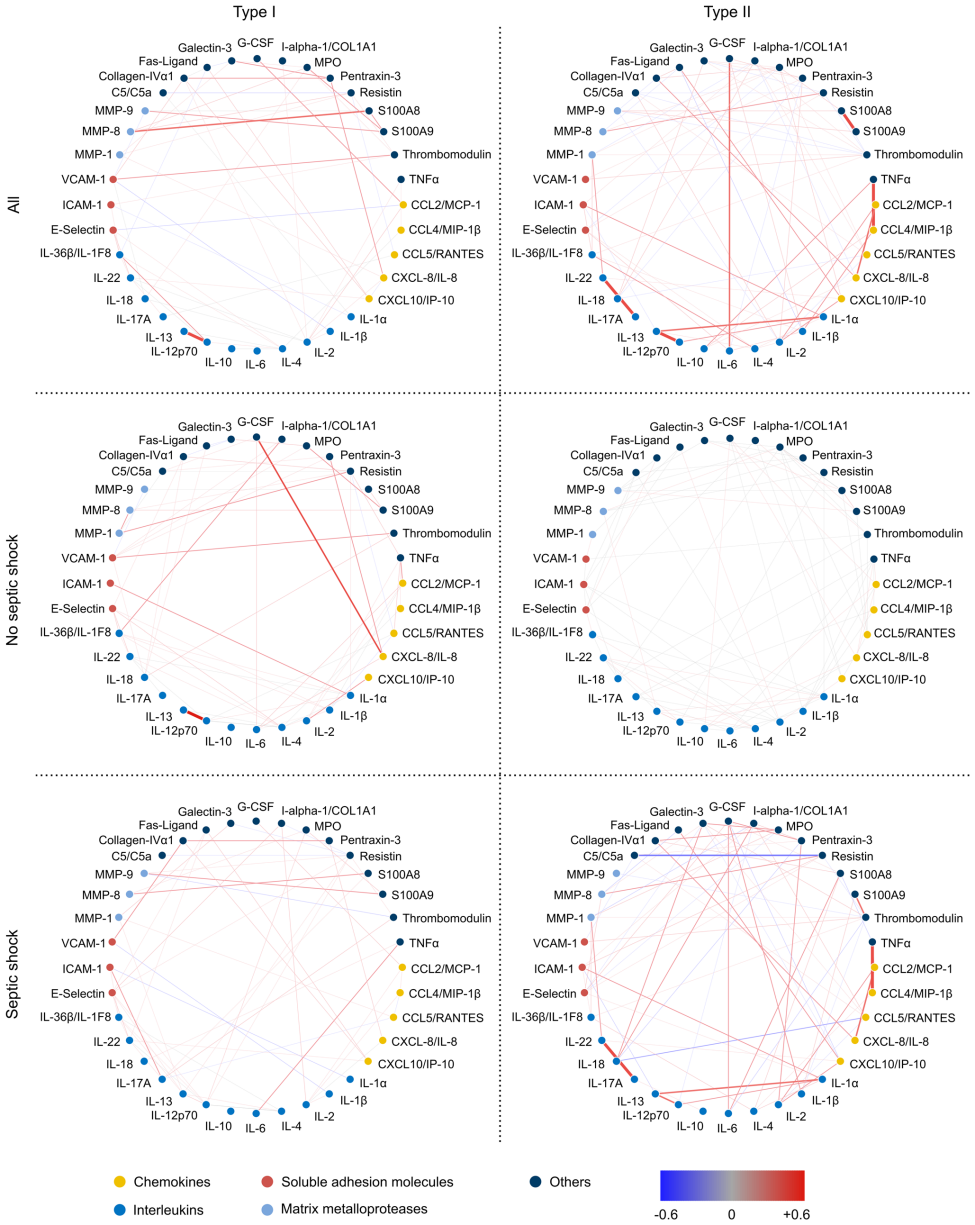
Type I and type II NSTIs display contrasting association networks. The colors of the circles indicate the categories of the analytes. The strength of the partial correlation between analytes is indicated by the color and the weight of the connection.

The specific biomarker panels identified in type I versus type II NSTIs as well as the septic shock profiles displayed by either type implied differential mechanisms underlying the skewed inflammatory responses. To gain further insights into this and to identify key response nodes, network connectivity analysis was applied to assess interactions among the markers in the discovery cohort data set (Figure 7 and Supplemental Figures 4 and 5). In general, we observed a more densely connected network in NSTIs in comparison with both control groups (Supplemental Figure 4). Whereas most analytes were disconnected in the controls, some analytes, such as pentraxin 3 and CXCL-8/IL-8, gained many interacting partners in NSTI cases. The results revealed that the differences in connectivity were related not only to the presence of unique connections, but also to the strength of the connections. Furthermore, the analyses revealed striking differences in connectivity patterns even between the different NSTI clinical phenotypes (Figure 7). The most pronounced differential connectivities between type I and type II NSTIs were noted for IL-6, IL-1α, and CCL4, all of which were stronger in type II. The analytes with a high number of connections (hubs) that displayed significant differential connectivity between septic shock and nonseptic shock were different for the 2 types of NSTIs. In type I cases, connections among the interleukins IL-1α, IL-4, and IL-17A were the most relevant (*q* < 0.002), while type II NSTIs displayed the most changes in other analytes, such as galectin 3, I-α-1/COL1A1, and thrombomodulin (*q* < 0.001) (Supplemental Table 9).

## Discussion

In this study, we identify a set of plasma biomarkers that discriminate between distinct clinical NSTI phenotypes. Robust profiles were defined for NSTI versus non-NSTI controls and type I and type II NSTIs as well as septic shock development. A key strength of the study is that it is based on the prospective multicenter NSTI patient cohort (the INFECT cohort), which is the largest available NSTI cohort, and it also includes an extensive biobank collected using harmonized standard operating procedures (24). To identify analytes with the highest predictive power to discriminate between different clinical phenotypes, a set of stringent statistical analyses was applied to the data set, including uni- and multivariate analyses with embedded resampling to account for unequal patient numbers in specific patient groups. The multivariate analyses included clinical parameters of age, sex, and SOFA score to assess their contributions to the identification of the different clinical phenotypes. The finding of unique predictive biomarker panels related to specific clinical phenotypes suggests differential underlying pathophysiological mechanisms. This was further strengthened by the connectivity analyses demonstrating differential marker-marker interactions as well as different key hubs (i.e., densely connected) in the specific clinical phenotype–linked networks.

The development of rapid diagnostic tools for NSTI, such as levels of disease-associated biomarkers, to support clinical decisions could increase the accuracy of early diagnosis, leading to swifter surgical exploration and treatment only when clinically indicated. However, to date, there are only a few studies of molecular biomarkers in NSTIs (22, 28–32), and these are limited to analyses of only a few markers. The comprehensive multiplex analysis of 36 analytes conducted here revealed, as expected, a greater systemic inflammatory response in NSTI patients than in noninfected patients (surgical controls). Less drastic changes were observed when NSTI patients were compared with the infected non-NSTI controls. This is in line with the non-NSTI cases having a severe soft-tissue infection, to the extent that they were initially suspected NSTIs, but in which no necrosis was found upon surgical exploration, and hence, greater similarity in the host response is reasonable. Notably, thrombomodulin emerged as a robust candidate for the discrimination of NSTI from non-NSTI, indicating its potential as a biomarker for soft-tissue necrosis. Although there are no published reports exploring thrombomodulin in soft-tissue infections, it has been linked to necrotizing pancreatitis (33). Further studies are needed to dissect the underlying mechanism leading to elevation of thrombomodulin and its role in NSTI and necrosis. Our data support that thrombomodulin is a biomarker of relatively early disease, as it was noted in patients showing only early signs of NSTI. However, these data need to be interpreted with caution, since classifications of early and late signs are based on patient chart notes and, because of this, potential bias cannot be excluded. Therefore, further studies are warranted, and it would be of value to assess samples already collected in the ambulance or the emergency department.

There were no differences between thrombomodulin levels in type I and type II, which is in agreement with previous reports demonstrating high levels of soluble thrombomodulin in bacterial infections regardless of the causative microorganism (34, 35). Moreover, elevated concentrations of thrombomodulin in blood have been reported in patients with sepsis (35–38). Such elevated levels were also detected in the sepsis cohort we included during the validation stage. Notably, thrombomodulin retained its discriminatory power for NSTIs. Finally, thrombomodulin has also been proposed as a biomarker for the prediction of mortality in patients with sepsis (35, 37) and septic shock (39). Although an association with mortality in NSTI was not noted in our study, a weak association with septic shock was identified.

Identification of biomarkers associated with septic shock in NSTI patients was a key focus of this study, as early identification of this complication is critical for optimal tailored patient management. The plasma inflammatory response profile indicated a septic shock signature that was dependent on the NSTI type. Three septic shock–associated markers, i.e., IL-6, G-CSF, and S100A8, were shared for both types. In the validation stage, we confirmed the discriminatory power of all 3 biomarkers for septic shock. Hence, this confirms their biomarker potential in NSTIs and likely also in other severe infectious diseases, such as sepsis. Additionally, we also explored biomarker signatures for major outcomes, such as death and amputation. We failed to identify a significant biomarker signature related to these outcomes, which may be due to our highly stringent analyses. It should also be noted that amputation as readout is associated with many confounders, such as praxis at the clinical site.

Early targeted antibiotic treatment of NSTIs is critical for the successful management of patients, and therefore, biomarkers for the discrimination of types I and II NSTIs could serve to accelerate the decision-making process in the clinics. In this study, CXCL10, IL-2, IL-10, IL-22, MMP-9, and Fas-ligand were identified as discriminatory biomarkers for type I and type II infections. Out of these 6 markers, MMP-9 was the only marker with higher concentrations in type I versus type II, whereas the rest were higher in type II. We sought to validate the predictive biomarker sets identified in the discovery cohort by in vitro stimulation experiments to model the type I and type II infections. The measurement of the selected analytes in media from the validation stimulations experiments showed higher levels of CXCL10, IL-2, IL-22, and Fas-ligand associated with type II, as compared with type I, bacterial stimulation. However, IL-10 responses in in vitro stimulations did not match the variation noted in patient plasma. This discordant result is likely due to the limitations in the in vitro assay failing to mimic the complex in vivo setting. Nonetheless, 4 out of 5 tested biomarkers corroborated the patient data and substantiated the association of specific biomarkers to the type of infection.

Among all tested biomarkers, CXCL10 displayed the strongest power to discriminate type II from type I NSTIs. Although this association was noted in both the discovery and the validation cohort, it was substantially more impressive in the discovery cohort. As there was a difference in the frequency of GAS type II cases between the 2 cohorts, a subanalysis including only type II GAS cases was performed and revealed an almost perfect differentiation from type I cases. Hence, the relevance of this biomarker is likely connected to GAS rather than to all type II infections. In line with this, our recent study using dual RNA-Seq analyses of processed tissue biopsies from NSTI patients revealed a higher expression of CXCL9, CXCL10, and CXCL11 in NSTI GAS type II infections versus type I (21). Moreover, IL-10 and IL-2 have been previously linked to severe and nonsevere GAS infections. IL-10 has been reported to be elevated during GAS infections and significantly higher in invasive versus noninvasive infections (40, 41). The frequency of IL-2–producing cells in circulation increased in patients with severe invasive GAS infections (42). Finally, we are not aware of reports that have measured Fas-ligand and IL-22 on serum or plasma linking their levels to type of infection, and therefore this study is the first, to our knowledge, to report their potential relevancy. Taken together, these findings underscore the need for in-depth studies at the species level. Such analyses are beyond the scope of this study, but are strongly warranted, particularly in type II NSTIs, which are predominantly also caused by other β-hemolytic streptococci, such as *S*. *dysgalactiae* (17).

Our results demonstrate a distinctive inflammatory profile in the different clinical phenotypes, likely resulting from pathogen-specific underlying mechanisms. This concept was further explored through differential connectivity analyses delineating the interconnections, and magnitudes thereof, for each plasma analyte. The results highlighted distinct networks and hubs dependent on the type of NSTI and septic shock. This analysis shifts the focus toward the relationships between analytes rather than on their levels, making it a useful tool in systems biology for investigating and understanding complex biological data (13, 43). The potential of personalized medicine in NSTIs has been emphasized in recent reports (12), and it is tempting to speculate that the hubs identified represent potential targets for interventions, as the associated network is more likely to be affected. It will be of interest in future studies to explore the role of these key hubs in pathophysiology and as therapeutic targets in NSTI.

In conclusion, in this study, we identified discriminatory biomarkers for NSTI and its clinical phenotypes: (a) soft-tissue necrosis (thrombomodulin); (b) type I versus type II NSTIs (MMP-9, CXCL10, IL-10); and (c) septic shock versus no shock (IL-6, G-CSF, and S100A8). These biomarkers are promising candidates for improved diagnosis and prognosis, which is highly anticipated in clinical practice to decrease the rate of misdiagnosed cases and improve therapeutic strategies in NSTIs.

## Methods

### Patient cohorts.

The study is based on clinical data and plasma samples from patients with NSTI (surgically confirmed) enrolled in the multicenter INFECT study. Samples were collected in 5 hospitals in Scandinavia: Blekingesjukhuset (Karlskrona, Sweden), Haukeland University Hospital, Karolinska University Hospital, Righospitalet (Copenhagen, Denmark), and Sahlgrenska University Hospital. Clinical data considered for analyses were recorded at the time of admission to the specialized hospital and were entered into a web-based electronic case report form (eCRF) by trial personnel. Patient characteristics and outcomes for the whole cohort have been reported in Madsen et al. (5). Of the available plasma samples from the INFECT cohort, 251 samples were considered for the discovery cohort (Figure 1). The size of this cohort was limited by technical availability, and samples were selected at random. Due to the lack of other NSTI cohorts with the associated biobank, the validation cohort consisted of a second set of plasma samples from the remaining patient samples from the INFECT study. The size of the validation cohort was determined based on technical availability (*n* = 60). Selection of samples prioritized type II NSTI samples, since only 21 remained available, and then 39 samples from type I NSTI patients were selected at random.

Two additional cohorts of 20 patients each were included as control groups for the discovery cohort. The non-NSTI patient samples were collected during the INFECT study and included patients with suspected NSTI who, after surgical examination, were diagnosed with less severe soft-tissue infections due to lack of necrotic tissue. The surgical controls included patients who had undergone elective surgery at Rigshospitalet for noninfectious conditions and who had no underlying diseases (22). These 2 control groups were matched in age and sex to the discovery NSTI cohort.

Finally, our study included an additional sepsis cohort of 24 patients (42% septic shock) to determine whether the panel is valid selectively for NSTI cases or would also apply to a broader sepsis patient group. Plasma samples of the sepsis cohort were collected at admission from patients with sepsis at the emergency clinic at the Karolinska University Hospital (Huddinge, Sweden). The size of this cohort was determined by sample availability.

### Measurement of potential biomarkers in plasma.

The plasma samples were prepared from blood collected at admission in EDTA-containing tubes and immediately aliquoted and frozen at –80°C. Concentrations in plasma of the selected list of analytes were determined using the bead-based Luminex multiplex immunoassay. Assays were performed according to the manufacturer’s protocol and acquired on a Luminex MAGPIX instrument using xPonent 4.0 software (Luminex). The measurements of the discovery cohort were done in 2 customized multiplex plates of 5 and 32 analytes (R&D Systems). The panel included chemokines (CCL2/MCP-1, CCL4/MIP-1β, CCL5/RANTES, CXCL-8/IL-8, CXCL10/IP-10), interleukins (IL-1α, IL-1β, IL-2, IL-4, IL-6, IL-10, IL-12p70, IL-13, IL-17A, IL-18, IL-22, IL-36β/IL-1F8), adhesion molecules (E-Selectin, ICAM-1, VCAM-1), matrix metalloproteases (MMP-1, MMP-8, MMP-9), and others (C5/C5a, collagen-IVα1, Fas-ligand, Galectin-3, G-CSF, I-α-1/COL1A1, MPO, Pentraxin-3, Resistin, S100A8, S100A9, thrombomodulin, and TNF-α). The initial panel included IL-1RA; however, this analyte was not included in the final analyses due to a high number of out of range (OOR) values (>30%).

For the validation cohort, only the most robust biomarkers identified in the discovery cohort were assessed. Two panels were measured in customized multiplex plates from R&D Systems (G-CSF, IL-6, S100A8, and thrombomodulin) and Thermo Fisher (MMP-9, CXCL10, IL-2, IL-10, IL-22, and Fas-ligand). The results from IL-22 were not included in the final analysis due to a high number of OOR values (>30%).

### Cell isolation for in vitro validation.

PBMCs were isolated from peripheral blood of healthy blood donors by Ficoll-Hypaque density gradient centrifugation (Lymphoprep, Axis-Shield) and were resuspended in complete RPMI media (RPMI 1640 [Life Technologies] supplemented with 10% FBS [Sigma-Aldrich], 2 mM l-glutamine [Thermo Fisher Scientific], and 25 mM HEPES [Thermo Fisher Scientific]). The cells were rested overnight at 4°C and were seeded on the day of the experiment at a concentration of 1 × 10^6^ cells/well in a 96-well plate.

### Bacterial strains.

The bacterial strains of GAS, *E*. *coli*, and *B*. *fragilis* are part of the INFECT biobank and were isolated from NSTI patients 2006 (type II NSTI caused by GAS) and 4011 (type I NSTI). *B*. *fragilis* was cultured inside an Oxoid 2.5 L jar (Thermo Fisher Scientific) with Oxoid AnaeroGen 2.5L sachets (Thermo Fisher Scientific). Bacterial strains were grown from a single colony in brain heart infusion (BHI) broth supplemented with 5% FCS at 37°C in an incubator without shaking overnight. After 16 hours, 3 ml of the cultures was collected, and new cultures were inoculated from the ON at an OD_600_ of 0.05. The bacterial cultures were grown until late exponential phase (GAS, OD ~1; *E*. *coli*, OD ~0.8; and *B*. *fragilis*, OD ~0.6), and 3 ml was collected for further processing.

### PBMC stimulation.

Collected bacterial cultures were centrifuged at 1600 *g* for 10 minutes, and the supernatant was collected and filtered with a 0.2 μm filter. The bacterial pellet was washed and resuspended in 1 ml PBS. The HK sample was prepared by incubation of the resuspended pellet at 75°C for 30 minutes. Stimulation of PBMCs was carried out with a mix of exponential and stationary samples dissolved in RPMI complete. HK samples for stimulation were diluted to an equivalent of a multiplicity of infection of 9, while a 1:200 dilution was used for stimulation with supernatants. The PBMCs were stimulated with samples of GAS, *E*. *coli*, *B*. *fragilis*, or a 1:1 mix of the latter two for 24 hours in a 5% CO_2_ incubator at 37°C. Two control stimulations were included: RPMI media with 10% PBS and RPMI with 10% BHI. Cell culture supernatants were collected by centrifugation at 500 *g* for 5 minutes. All samples were frozen at –20°C for 16 hours and then transferred to –80°C for long-term storage. In total, 5 independent biological replicates were carried out.

Concentrations of the analytes of interest (CXCL10, Fas-ligand, IL-2, IL-10, and IL-22) in the cell culture media were determined using the bead-based Luminex multiplex customized plates (R&D Systems) or the IL-22 ELISA Kit (Peprotech) according to the manufacturer’s instructions. Prior to measurement, samples were thawed on ice and centrifuged again at 500 *g* for 5 minutes.

### Handling of censored data.

Imputation of censored data was only carried out in the discovery cohort. Some of the measured values from the multiplex analyses were found to be below or above the measurable range (OOR; Supplemental Table 10), and to generate a complete set of data, these values were imputed. Censored values from cytokines with only one missing value were substituted directly to half the minimal value (left censored) or the maximal plus 20% (right censored). For all other cytokines, the imputation was performed using the method proposed previously (44). The imputation for cytokines with double-censored data (i.e., both left and right censored) was carried out in 2 sequential imputation steps. First, the data were made left censored by setting the censored values above the range to the maximum observed value and then performing imputation of the left-censored data. Then the right-censored values were set back to be censored and imputed.

### Statistics.

Significant differences in the clinical data between cohorts or between subsets were tested by Mann-Whitney *U* test or Fisher’s exact test. Wilcoxon’s matched-pairs signed-rank test was used to test the differences in the in vitro stimulations. Statistical testing of the multiplex results was performed using the Kruskal-Wallis test, followed by Dunn’s post hoc test in the case of a 3-group comparison (i.e., NSTI vs. non-NSTI vs. surgical controls) or Mann-Whitney *U* test for comparison of 2 groups of samples.

To consider unequal group sizes in the discovery cohort, we used a resampling approach to make statistical comparisons. The groups to be compared were made the same size by randomly sampling *k* samples from each group, where *k* was chosen to be equal to 90% the size of the smallest group, and then performing statistical testing on these equally sized subgroups. The overall procedure was repeated 10^4^ times. We deemed robust and generalizable only those comparisons that were found to be significant in 95% of the runs. The adjustment of *P* values was done using the Benjamini-Hochberg adjustment method (45). Statistical tests for biomarkers linked to the risk of amputation excluded patients with NSTI in nonamputable sites (i.e., neck, abdomen, and thorax) or who had undergone amputation before admission. Statistical comparisons of the results from the validation cohort did not use the resampling method, and the Mann-Whitney *U* tests were carried out in a standard manner.

### ROC analysis.

In addition to the statistical comparison of the biomarker’s levels between different subsets of patients, the diagnostic ability of each marker was tested by ROC analysis. The optimal threshold was selected as the point closest to the top-left part of the plot, which represents perfect sensitivity and specificity. The differences in group sizes in the discovery cohort were also considered for this test, and therefore the resampling methodology explained above also applied to this analysis. The results from the 10^4^ curves were assessed by calculating the mean of all outcomes.

### RF.

RF models (46) for the discovery cohort values were built using 10^5^ decision trees, and 6 random cytokines were picked at every split selection. To measure the importance of every cytokine in the classification model, mean decrease Gini index was used. Statistical significance was calculated by the means of permutation test using 100 permutations of the original data sets as implemented in the rfPermute package.

### Logistic regression.

For the validation cohort, the association between the selected analytes and different outcomes was assessed by calculating odds ratios based on logistic regression analysis. All analytes’ concentrations were transformed with log_2_ before the generation of the model. The odds ratios were obtained by exponentiation of the model coefficients. Multivariate logistic regressions were performed to correct for sex and age.

### Network analyses.

Protein association networks were built using the context likelihood of relatedness based on correlation algorithm (PCLRC), which was first introduced to reconstruct metabolite correlation networks and shown to be robust against variation in sample size and noise (47). In the present study, we used pairwise partial correlation among proteins measured on the different patient groups as a weighted measure of analyte association to reduce the chances of false indirect associations. PCLRC gives the probability of likelihood of occurrence of a relationship between the cytokines. Associations with probability weights of more than 0.95 were retained in the analysis. Cytokine-association networks were built for different patient groups and compared as detailed below.

### Differential connectivity analysis.

Differential connectivity was used to compare the cytokine association networks of different patient groups and to highlight cytokines whose patterns of association vary. Differential connectivity analysis has been successful in investigating potential molecular mechanisms underlying different conditions in biological systems (48). The connectivity for each node (i.e., protein) in the network is defined as the summation of the absolute values of the weights of all the edges associated with the given node, thereby accounting for both the number of connections and the weight of those connections. Thus, for *i*th cytokine, cytokine connectivity *X_i_* is given by the following:

(Equation 1)
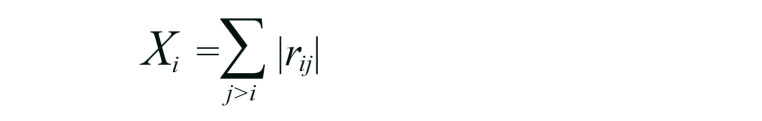


where r is the correlation, as defined by the PCLRC algorithm, between cytokines *i* and *j*. The differential connectivity (*ΔX_i_*) of the *i*th cytokine in networks from group 1 (G1) and group 2 (G2) can be given by the following:

(Equation 2)
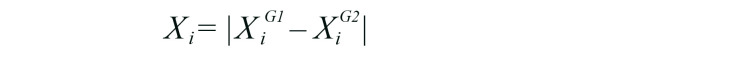


The statistical significance of the observed differential connectivity for each cytokine was established by using a permutation test (48, 49). The procedure included the independent permutation of the values of every protein repeated 10^3^ times with the intention of deriving a probability of the observation in the form of a *P* value.

### Software.

All statistical tests included in this paper were performed in R, version 3.6 (50), or GraphPad Prism, version 8.2.0, for Windows. Kruskal-Wallis and Mann-Whitney *U* tests were performed using the R *stats* package, and the post hoc Dunn’s test was performed using the FSA package (51). The ROC tests were performed with the R package pROC (52). The logistic regression was perform using *glm*, and the confidence intervals were obtained with *confint*, both functions from the package *stats*. The RF models were built using the R package *rfPermute* (53). The R code for PCLRC is available at the Waganingen University Laboratory of Systems and Synthetic Biology website (www.systemsbiology.nl) under the software tab. Finally, Fisher’s exact test for comparison of clinical data and Wilcoxon’s matched-pairs signed-rank test for the statistical comparison of in vitro results were performed using GraphPad Prism, version 8.2.0.

### Study approval.

The multicentre INFECT study is registered at ClinicalTrials.gov (NCT01790698). The study was approved by national ethics committees, including the Regional Ethical Review Board at the Karolinska Institute in Stockholm, Sweden (ethics permits 2012/2110-31/2), the regional Ethical Review Board at the National Committee on Health Research Ethics in Copenhagen (ref. no. 1211709, including amendment 4:61050; regional ethics committee H-2-2014-071), the Regional Ethics Committee in Gothenburg (ref. no. 930-12), and the Regional Ethics Committee in Vest, Norway (ref. no. 2012/2227/REK VEST). Use of sepsis samples included in the study was approved by the Regional Ethical Board in Stockholm (2017/1358-31) All studies were conducted in accordance with the Declaration of Helsinki. All samples were pseudonymized. All patients or their legal guardians provided informed consent prior to enrollment and sample collection.

### Data and materials availability.

All data associated with this study are available in the main text or the supplementary materials. The raw data with and without imputed values are available at Dryad (DOI: 10.5061/dryad.f1vhhmgw4; https://datadryad.org/stash/share/zclF2y-NfdaaSAKY-6N02TmWJhd9oONAayDySmCTzy8).

## Author contributions

LMPM, ER, TB, SS, MS, and ANT conceived the project. LMPM, ER, TB, MBM, KS, CU, MBH, PA, MN, OH, SS, MS, and ANT established the protocols for handling clinical material and performing measurements. ER, TB, MBM, MBH, OH, KS, CU, PA, MN, and SS provided resources. LMPM, ER, and ML performed experiments. LMPM was responsible for data curation. LMPM, ER, SJ, TB, and ES carried out formal analysis of the results. LMPM and SJ prepared visualizations. LMPM and ANT wrote the original draft. LMPM, ER, SJ, TB, MBM, KS, CU, MBH, PA, MN, OH, ML, VAPMDS, ES, SS, MS, and ANT reviewed and edited the manuscript. TB, VAPMDS, ES, SS, MS, and ANT supervised the project. OH, VAPMDS, SS, KS, MS, and ANT administered the project, including funding acquisition.

## Acknowledgment

We thank the patients and relatives for their consent to participate and the clinical staff at the study sites for their invaluable contribution. Thanks are due also to Kristin Rye at the University of Bergen, Norway, for expert technical assistance performing the Luminex analysis. The microbiological laboratories of the participating hospitals are gratefully acknowledged for performing routine culture and identification of microbial etiologies. This work was supported by the Center for Innovative Medicine (CIMED) and Region Stockholm (no. 20180058); the Swedish Research Council (2018-02475); the European Union Seventh Framework Programme (FP7/2007-2013) under the grant agreement 305340 (INFECT project); the Swedish Governmental Agency for Innovation Systems (VINNOVA), Innovation Fund Denmark, and the Research Council of Norway under the frame of NordForsk (project no. 90456, PerAID); the Swedish Research Council, Innovation Fund Denmark, the Research Council of Norway, the Netherlands Organisation for Health Research and Development (ZonMW), and DLR Federal Ministry of Education and Research, under the frame of ERA PerMed (project 2018-151, PerMIT); and the Swedish Children’s Cancer Foundation (TJ2018-0128).

## Supplementary Material

Supplemental data

Trial reporting checklists

ICMJE disclosure forms

## Figures and Tables

**Table 1:**
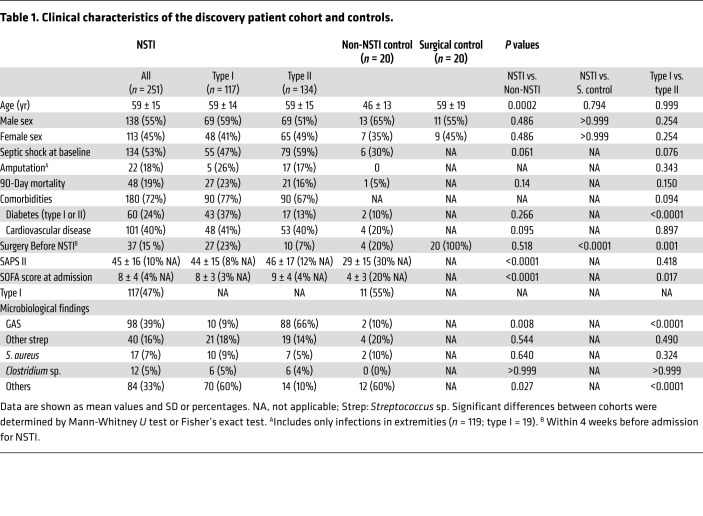
Clinical characteristics of the discovery patient cohort and controls.

## Version 1 07/15/2021

Electronic publication
