# Supplementary material for: Discriminatory plasma biomarkers predict specific clinical phenotypes of necrotizing soft-tissue infections
Source: J Clin Invest. 2021 Jul 15;131(14):e149523. doi: 10.1172/JCI149523 (PMC8279592; doi:10.1172/JCI149523)
Supplement: Supplemental data [file jci-131-149523-s067.pdf]

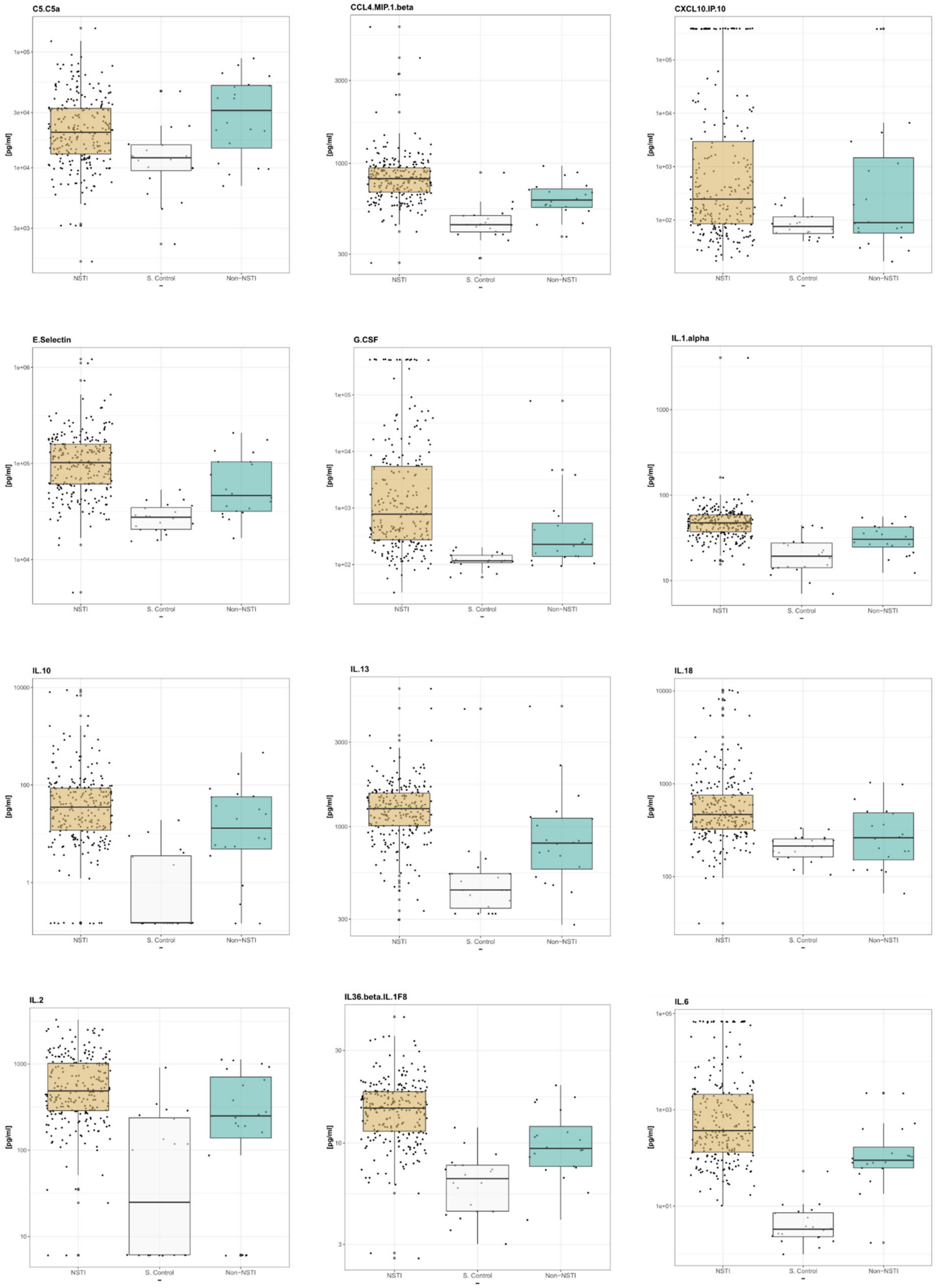

Continue in the next page.

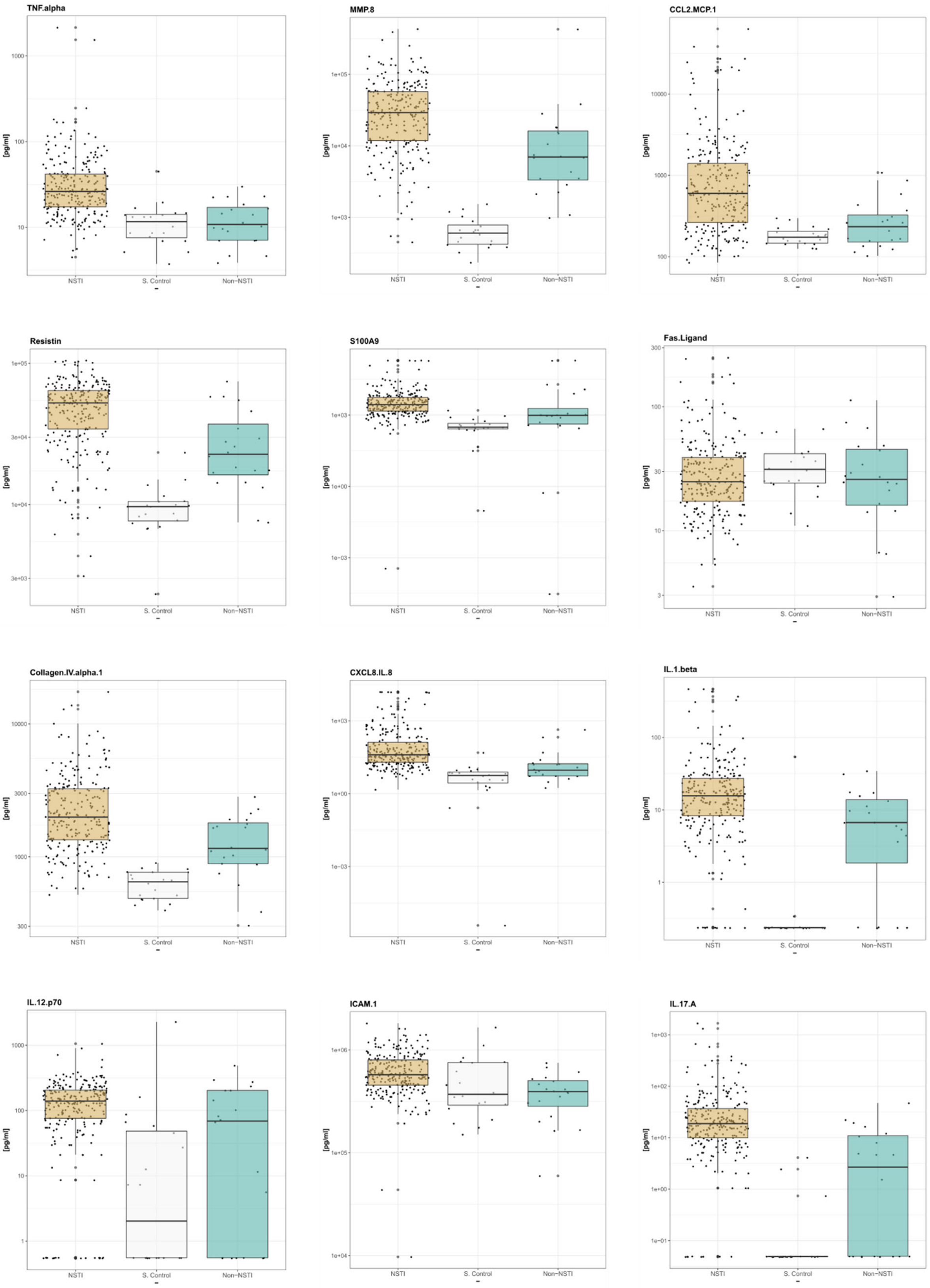

Continue in the next page.

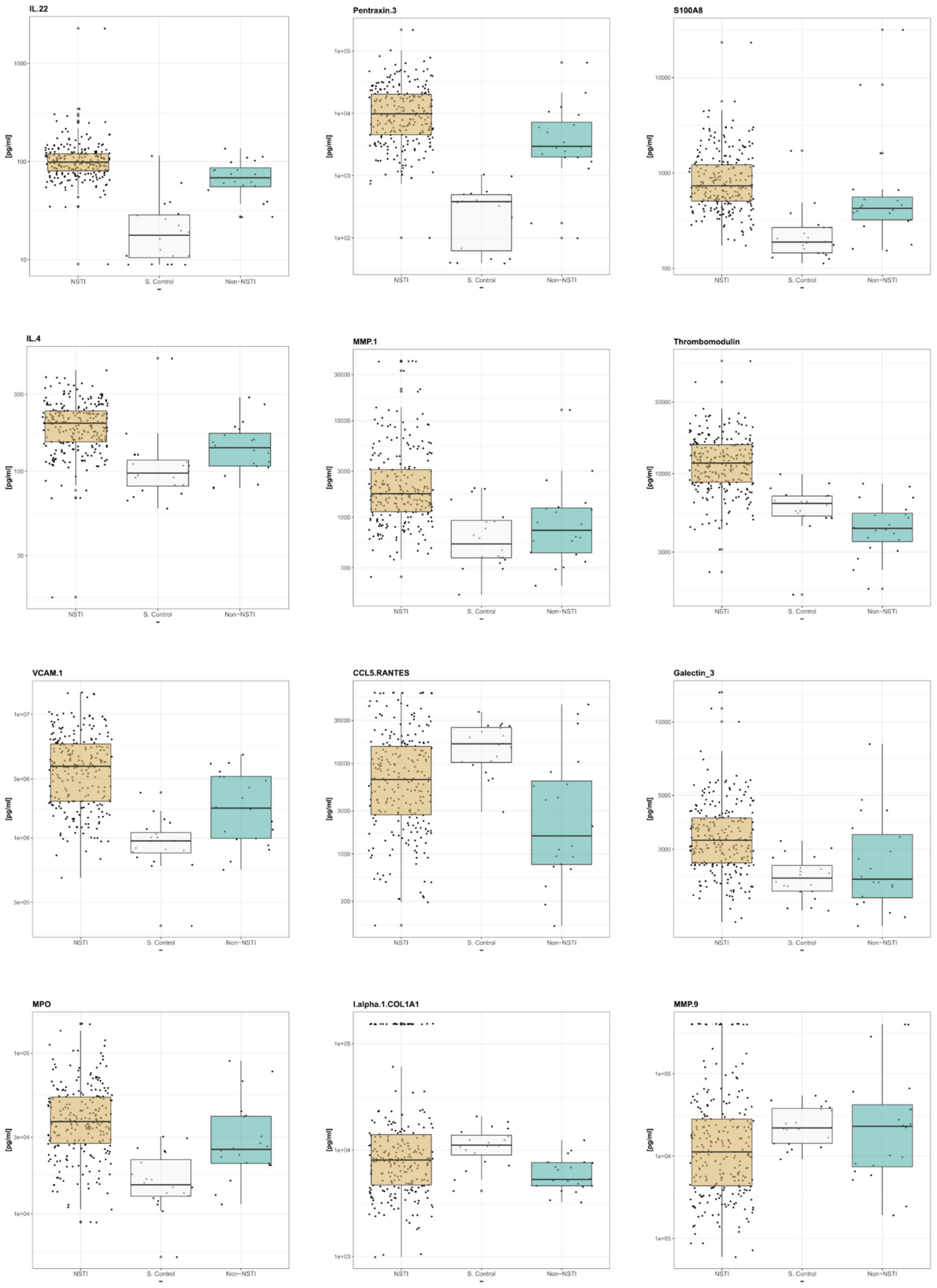
IL.22
1000
100
10
[pg/ml]
NSTI
S. Control
Non−NSTI
Pentraxin.3
1e+05
1e+04
1e+03
1e+02
S100A8
10000
1000
100
IL.4
300
100
30
MMP.1
30000
10000
3000
1000
300
Thrombomodulin
30000
10000
3000
VCAM.1
1e+07
3e+06
1e+06
3e+05
CCL5.RANTES
30000
10000
3000
1000
300
Galectin_3
10000
5000
3000
MPO
1e+05
3e+04
1e+04
I.alpha.1.COL1A1
1e+05
1e+04
1e+03
MMP.9
1e+05
1e+04
1e+03

◄ **Figure S1. Concentrations of the analytes in the discovery cohort, surgical controls and non-NSTI.** The boxplots depict the median and the first and third quartiles, whisker extends to the smallest and largest values or 1.5 times the inter-quartile range at most. NSTI: Necrotizing soft tissue infection, S. Control: Surgical controls, Non-NSTI: Suspected NSTI but no necrotic tissue found upon surgical exploration.

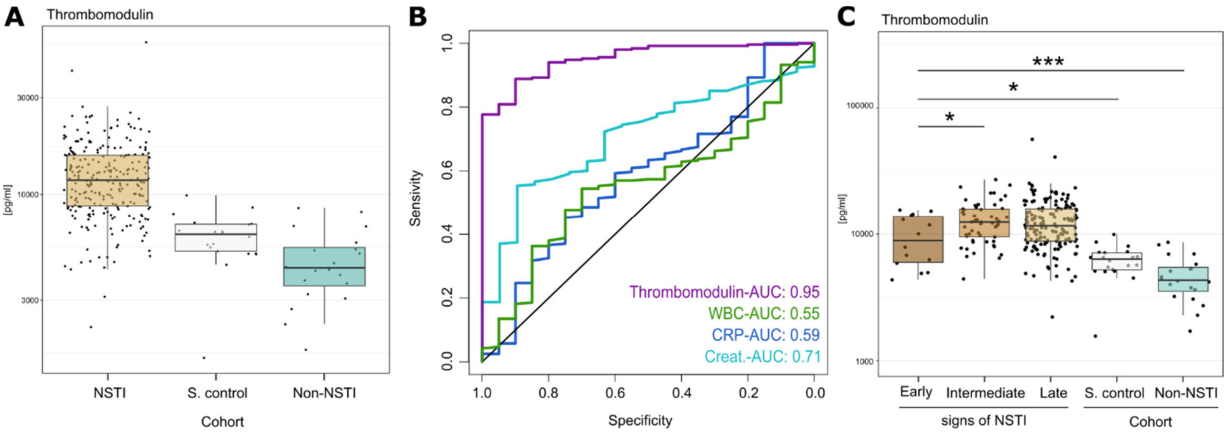

Figure S2. Significantly elevated concentration of Thrombomodulin in the NSTI discovery cohort as compared to both the surgical control and non-NSTI control cohorts. (A) Measured levels of thrombomodulin. **(B)** ROC curves of thrombomodulin and selected clinical markers. The legend shows the AUC (Area under the curve) of each curve. **(C)** NSTI patients were categorized in three groups based on NSTI signs**:** Early (severe pain, in need of opioids), intermediate (skin bullae or skin bruising) and late (skin purple/black discoloration, skin anesthesia, palpable gas (crepitus) or gas visualized on radiology) signs of NSTI. Statistical testing was performed using Kruskal-Wallis (KW) test followed by Dunn's post hoc test. The resulting p-value of the KW test was $7.62x10^{-15}$ and the significant differences between patients with early signs and the different groups are shown with stars (*= p-value < 0.05, and ***= p-value<0.005). The boxplots depict the median and the first and third quartiles, whisker extends to the smallest and largest values or 1.5 times the inter-quartile range at most. NSTI: Necrotizing soft tissue infection, S. controls: Surgical controls, Non-NSTI: Suspected NSTI but no necrotic tissue found upon surgical exploration, WBC: white blood cells, CRP: C-reactive protein.

Table S1. Results of the ROC analyses and the random forest model discriminating NSTI (discovery cohort) and non-NSTI. The ROC results are shown as the mean values of the iterations. Thresholds are given as pg/ml. The p-values of the random forest results were calculated using 100 permutations of the original dataset. (*Continues in the next page*)

	Receiver operating characteristic					Random Forest							
						Only proteins				with Clinical variables			
Analyte	AUC	[95% CI]	Threshold	Sn	Sp	MDA	p-val	MDG	p-val	MDA	p-val	MDG	p-val
CCL2/MCP-1	0.77	[0.61-0.93]	376.39	0.79	0.71	18.87	1.00	0.43	1.00	14.60	1.00	0.25	1.00
CCL4/MIP-1β	0.77	[0.62-0.93]	727.39	0.78	0.72	26.45	1.00	0.34	1.00	23.16	1.00	0.17	1.00
CCL5/RANTES	0.68	[0.5-0.86]	3944.64	0.63	0.75	63.37	**0.03**	1.79	0.13	73.29	**0.01**	1.33	**0.04**
CXCL-8/IL-8	0.82	[0.67-0.96]	20.10	0.80	0.80	82.91	0.32	2.02	**0.01**	56.38	0.44	1.00	**0.03**
CXCL10/IP-10	0.61	[0.42-0.8]	152.17	0.56	0.72	39.35	0.86	0.47	1.00	29.20	0.89	0.26	0.99
IL-1α	0.79	[0.64-0.94]	40.81	0.76	0.73	44.35	0.99	0.69	0.81	20.37	1.00	0.26	1.00
IL-1β	0.72	[0.55-0.89]	11.32	0.69	0.71	32.58	0.99	0.41	1.00	33.93	0.90	0.24	1.00
IL-2	0.68	[0.5-0.86]	311.64	0.61	0.77	59.79	0.80	1.15	0.21	31.93	0.95	0.74	0.10
IL-4	0.74	[0.57-0.91]	168.42	0.73	0.71	25.96	1.00	0.36	1.00	29.11	0.99	0.22	1.00
IL-6	0.74	[0.58-0.91]	146.03	0.74	0.77	30.89	1.00	0.45	1.00	25.81	0.99	0.31	1.00
IL-10	0.64	[0.45-0.82]	22.93	0.59	0.72	36.92	0.98	0.59	0.98	20.72	0.95	0.23	1.00
IL-12p70	0.65	[0.46-0.84]	92.97	0.62	0.74	50.95	0.90	0.61	0.92	52.84	0.66	0.38	0.75
IL-13	0.75	[0.58-0.92]	1012.96	0.72	0.79	47.89	0.88	0.65	0.87	34.21	0.90	0.33	0.90
IL-17A	0.81	[0.67-0.96]	9.83	0.74	0.82	61.77	0.71	1.68	0.07	57.75	0.46	1.09	**0.02**
IL-18	0.73	[0.56-0.9]	346.26	0.66	0.77	55.42	0.36	0.97	0.63	20.87	0.73	0.36	0.87
IL-22	0.76	[0.6-0.92]	83.65	0.74	0.75	65.21	0.84	0.87	0.46	26.68	1.00	0.22	1.00
IL-36β/IL-1F8	0.77	[0.61-0.93]	11.62	0.74	0.78	38.57	1.00	0.44	1.00	32.73	0.96	0.25	1.00
E-Selectin	0.71	[0.54-0.88]	67632.87	0.64	0.77	18.94	1.00	0.38	1.00	6.82	0.95	0.16	1.00
ICAM-1	0.78	[0.63-0.93]	489976.27	0.74	0.74	93.12	0.06	1.28	0.27	31.26	0.48	0.41	0.75
VCAM-1	0.77	[0.61-0.92]	2992116.00	0.76	0.69	26.32	0.76	0.76	0.84	12.91	0.93	0.27	0.99
MMP-1	0.78	[0.62-0.94]	1294.94	0.79	0.74	105.17	**0.02**	2.11	**0.04**	78.36	**0.03**	1.29	**0.01**
MMP-8	0.78	[0.62-0.94]	16814.91	0.78	0.74	40.65	0.90	0.97	0.54	26.08	0.92	0.60	0.33
MMP-9	0.60	[0.41-0.79]	17904.72	0.60	0.67	26.01	0.87	0.41	1.00	19.39	0.91	0.25	1.00
C5/C5a	0.60	[0.41-0.8]	30650.78	0.58	0.74	28.39	0.15	0.76	0.85	32.52	0.06	0.58	0.54
Collagen-IVα1	0.73	[0.57-0.9]	1625.95	0.70	0.71	17.81	0.99	0.42	1.00	10.21	0.99	0.28	1.00
Fas-Ligand	0.55	[0.35-0.74]	26.12	0.63	0.57	46.23	0.55	0.70	0.89	44.66	0.32	0.38	0.85
Galectin-3	0.70	[0.52-0.88]	2704.71	0.64	0.80	53.72	0.19	1.58	0.09	57.85	0.09	1.45	**0.01**
G-CSF	0.71	[0.54-0.89]	407.10	0.68	0.74	34.56	0.94	0.46	1.00	25.12	0.94	0.22	1.00
I-alpha-1/COL1A1	0.65	[0.46-0.84]	7385.20	0.78	0.60	42.00	0.29	0.50	1.00	33.83	0.34	0.29	0.99

MPO	0.67	[0.48-0.85]	30100.62	0.68	0.74	30.53	0.80	0.46	1.00	20.06	0.81	0.28	1.00
Pentraxin-3	0.73	[0.56-0.9]	5726.70	0.69	0.75	12.05	1.00	0.47	1.00	9.11	1.00	0.30	0.97
Resistin	0.77	[0.61-0.93]	34604.01	0.73	0.79	38.53	0.71	0.70	0.93	15.00	0.79	0.22	1.00
S100A8	0.72	[0.54-0.9]	547.20	0.74	0.74	21.63	1.00	1.14	0.16	24.16	0.99	0.85	0.06
S100A9	0.70	[0.51-0.9]	1249.99	0.74	0.82	65.64	**0.05**	1.81	0.08	47.80	0.08	1.28	**0.04**
Thrombomodulin	0.95	[0.89-1]	7566.85	0.92	0.89	217.09	**0.01**	6.36	**0.01**	111.07	**0.01**	2.54	**0.01**
TNFα	0.84	[0.72-0.97]	18.50	0.79	0.77	62.90	0.87	1.69	**0.03**	54.80	0.68	0.80	0.06
Age	NA	NA	NA	NA	NA	NA	NA	NA	NA	28.14	0.15	0.59	0.51
Sex	NA	NA	NA	NA	NA	NA	NA	NA	NA	-4.59	0.80	0.01	1.00
SOFA	NA	NA	NA	NA	NA	NA	NA	NA	NA	29.43	0.59	0.64	0.06
SS	NA	NA	NA	NA	NA	NA	NA	NA	NA	-0.51	0.87	0.03	0.48
Type	NA	NA	NA	NA	NA	NA	NA	NA	NA	-4.19	0.98	0.02	0.94
WBC	0.58	[0.38-0.78]	16.64	0.58	0.71	NA	NA	NA	NA	2.05	0.96	0.17	1.00
CRP	0.59	[0.4-0.79]	271.16	0.60	0.66	NA	NA	NA	NA	56.92	**0.02**	0.86	0.12
Creatinine	0.71	[0.53-0.89]	105.29	0.67	0.77	NA	NA	NA	NA	17.31	0.50	0.24	0.99

AUC: Area under the curve, CI: Confidence interval, Sn: Sensitivity, Sp: Specificity, MDA: Mean decrease accuracy, MDG: Mean decrease gini; SOFA: Sequential organ failure assessment score, SS: Septic shock, WBC: white blood cells, CRP: C -reactive protein, Type: microbiological classification of NSTI.

Table S2. Results of the ROC analyses and random forest model discriminating type I and type II NSTI in the discovery cohort. The ROC results are presented as the mean values of the iterations. Thresholds are given as pg/ml. The p-values of the random forest results were calculated using 100 permutations of the original dataset. (*Continues in the next page*)

						Random Forest							
	Receiver operating characteristic					Only proteins				with Clinical variables			
Analyte	AUC	[95% CI]	Threshold	Sn	Sp	MDA	p-val	MDG	p-val	MDA	p-val	MDG	p-val
CCL2/MCP-1	0.59	[0.51-0.66]	645.84	0.60	0.56	34.59	**0.04**	2.70	0.99	22.74	0.14	2.25	0.91
CCL4/MIP-1β	0.59	[0.51-0.66]	807.17	0.57	0.59	58.42	**0.02**	2.66	1.00	42.21	**0.03**	2.18	0.90
CCL5/RANTES	0.53	[0.45-0.6]	7411.44	0.50	0.58	3.88	0.26	2.37	1.00	1.34	0.36	1.96	1.00
CXCL-8/IL-8	0.51	[0.43-0.59]	52.10	0.64	0.46	54.46	**0.04**	2.75	1.00	52.60	**0.02**	2.43	0.86
CXCL10/IP-10	0.83	[0.78-0.89]	216.01	0.79	0.79	369.04	**0.01**	19.89	**0.01**	321.92	**0.01**	15.88	**0.01**
IL-1α	0.65	[0.58-0.72]	50.20	0.70	0.56	52.35	**0.03**	2.25	1.00	47.70	**0.02**	1.94	1.00
IL-1β	0.52	[0.44-0.6]	13.79	0.46	0.62	36.04	**0.02**	2.34	1.00	26.95	**0.03**	2.02	0.99
IL-2	0.74	[0.67-0.8]	523.28	0.75	0.67	132.37	**0.01**	6.88	**0.01**	115.87	**0.01**	5.61	**0.01**
IL-4	0.65	[0.58-0.72]	200.11	0.67	0.60	57.07	**0.01**	2.54	0.99	44.48	**0.03**	2.03	0.94
IL-6	0.61	[0.53-0.68]	451.85	0.62	0.56	31.78	**0.04**	1.95	1.00	23.63	0.10	1.69	1.00
IL-10	0.70	[0.63-0.77]	38.56	0.73	0.66	138.72	**0.01**	5.96	**0.02**	117.95	**0.01**	4.63	**0.01**
IL-12p70	0.66	[0.58-0.73]	165.10	0.75	0.53	79.49	**0.01**	3.19	0.50	75.22	**0.01**	2.72	0.25
IL-13	0.61	[0.54-0.69]	1280.83	0.62	0.57	30.65	0.08	1.97	1.00	21.10	0.06	1.58	1.00
IL-17A	0.67	[0.6-0.74]	19.83	0.67	0.61	106.05	**0.01**	3.71	0.27	89.56	**0.01**	3.08	0.18
IL-18	0.60	[0.53-0.68]	471.26	0.63	0.60	8.50	0.17	2.05	1.00	-2.71	0.48	1.57	1.00
IL-22	0.73	[0.66-0.8]	104.19	0.77	0.63	152.12	**0.01**	6.81	**0.01**	135.53	**0.01**	5.46	**0.01**
IL-36β/IL-1F8	0.65	[0.58-0.73]	14.89	0.61	0.66	71.62	**0.01**	2.41	1.00	53.63	**0.01**	1.86	1.00
E-Selectin	0.67	[0.6-0.74]	109952.76	0.68	0.61	74.10	**0.01**	3.42	0.65	58.02	**0.01**	2.78	0.61
ICAM-1	0.62	[0.55-0.7]	595130.42	0.67	0.59	40.08	**0.03**	2.33	1.00	52.16	**0.03**	2.27	0.99
VCAM-1	0.65	[0.57-0.72]	3490787.00	0.57	0.67	50.67	**0.02**	3.68	0.67	59.37	**0.02**	3.24	0.39
MMP-1	0.56	[0.48-0.64]	1679.64	0.63	0.55	43.03	**0.02**	3.23	0.97	30.76	0.06	2.09	1.00
MMP-8	0.52	[0.44-0.59]	27284.41	0.60	0.47	40.52	0.07	3.16	0.88	34.77	**0.05**	2.59	0.68
MMP-9	0.71	[0.64-0.78]	8858.61	0.77	0.61	139.42	**0.01**	5.92	**0.01**	112.09	**0.01**	4.17	**0.02**
C5/C5a	0.51	[0.43-0.59]	20808.04	0.47	0.61	29.15	0.08	2.69	1.00	30.95	0.08	2.14	1.00
Collagen-IVα1	0.62	[0.54-0.69]	2011.76	0.64	0.61	13.23	0.26	2.83	0.98	-1.30	0.45	2.19	1.00
Fas-Ligand	0.75	[0.69-0.82]	26.15	0.76	0.68	173.75	**0.01**	8.16	**0.01**	160.03	**0.01**	6.78	**0.01**
Galectin-3	0.52	[0.44-0.6]	3538.70	0.45	0.65	17.74	0.18	2.37	1.00	21.67	0.10	2.06	1.00

G-CSF	0.68	[0.61-0.75]	987.78	0.70	0.60	79.39	**0.02**	2.98	0.93	72.78	**0.01**	2.58	0.60
I-alpha-1/COL1A1	0.66	[0.59-0.73]	7214.79	0.58	0.71	16.78	0.13	3.01	1.00	21.27	0.10	2.55	0.93
MPO	0.52	[0.44-0.6]	37339.36	0.56	0.51	39.43	**0.04**	2.63	1.00	20.85	0.06	1.83	1.00
Pentraxin-3	0.61	[0.53-0.68]	9159.10	0.59	0.62	42.35	0.06	2.56	1.00	45.98	**0.02**	2.34	0.95
Resistin	0.54	[0.46-0.61]	54190.86	0.59	0.51	12.62	0.17	2.50	1.00	9.84	0.17	1.99	1.00
S100A8	0.63	[0.56-0.71]	732.57	0.63	0.61	50.82	**0.03**	2.15	1.00	44.01	**0.04**	1.80	1.00
S100A9	0.59	[0.52-0.67]	3596.50	0.52	0.67	54.82	**0.02**	3.20	0.94	34.85	0.06	2.18	1.00
Thrombomodulin	0.52	[0.44-0.6]	11441.51	0.50	0.56	-9.44	0.64	1.89	1.00	-18.07	0.80	1.44	1.00
TNFα	0.63	[0.56-0.71]	26.75	0.66	0.60	44.68	**0.04**	2.11	1.00	32.18	0.07	1.67	1.00
Age	NA	NA	NA	NA	NA	NA	NA	NA	NA	-0.42	0.42	1.27	1.00
Sex	NA	NA	NA	NA	NA	NA	NA	NA	NA	-3.10	0.47	0.18	0.99
SOFA	NA	NA	NA	NA	NA	NA	NA	NA	NA	2.87	0.29	1.12	1.00
SS	NA	NA	NA	NA	NA	NA	NA	NA	NA	1.33	0.26	0.14	0.98
WBC	0.59	[0.51-0.67]	14.08	0.50	0.67	NA	NA	NA	NA	33.00	0.08	1.97	1.00
CRP	0.64	[0.57-0.72]	290.74	0.61	0.61	NA	NA	NA	NA	58.37	**0.01**	3.07	0.40
Creatinine	0.59	[0.51-0.67]	134.04	0.56	0.63	NA	NA	NA	NA	3.52	0.38	1.56	1.00

AUC: Area under the curve, CI: Confidence interval, Sn: Sensitivity, Sp: Specificity, MDA: Mean decrease accuracy, MDG: Mean decrease gini; SOFA: Sequential organ failure assessment score, SS: Septic shock, WBC: white blood cells, CRP: C -reactive protein.

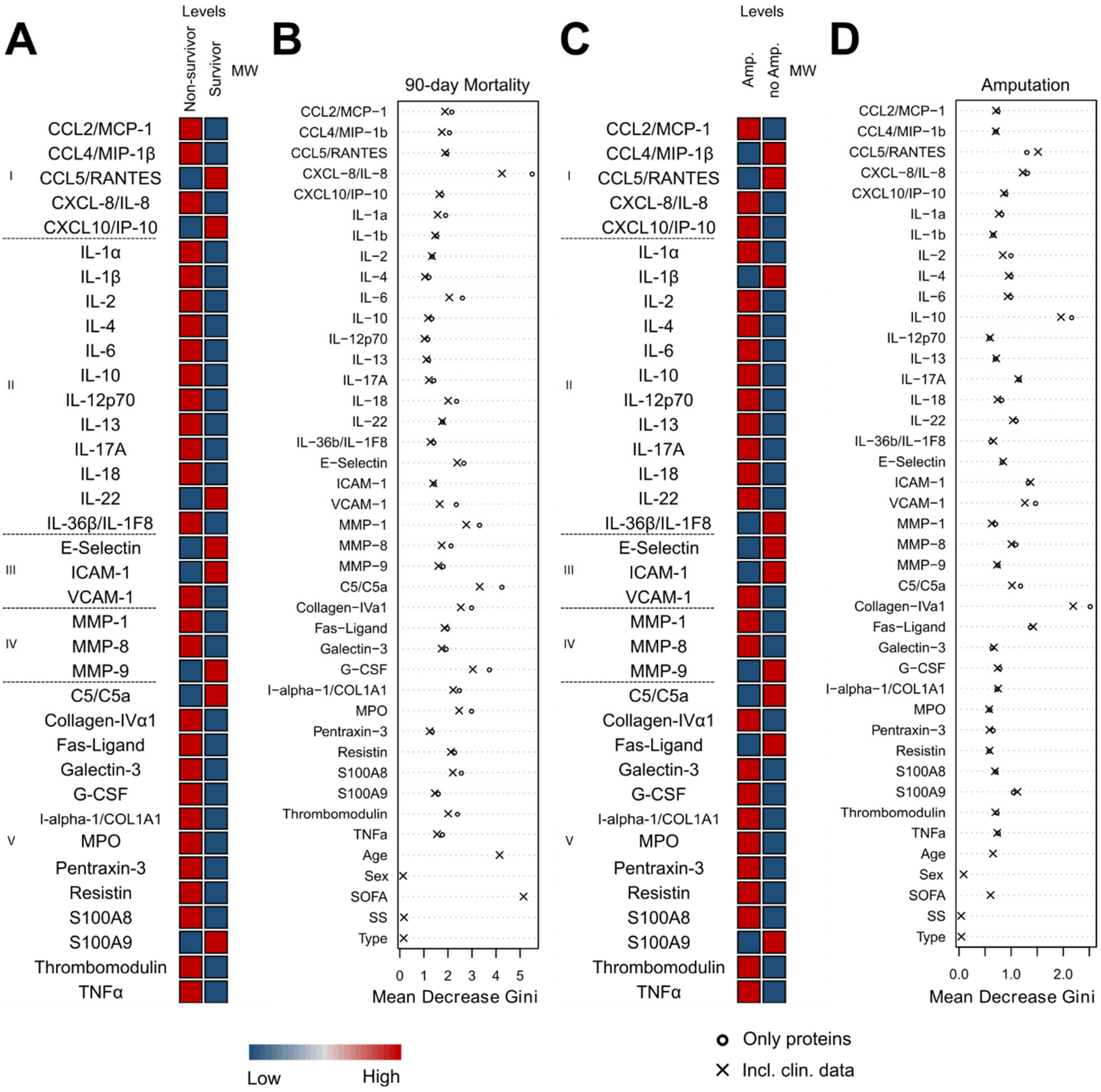

Figure S3. None of the measured analytes displayed significant differences for prediction of mortality or amputation in the discovery cohort. (A and C) The median protein levels in each group are depicted in the heatmap. The measured proteins are divided by categories: I-Chemokines, II-Interleukins, III-Soluble adhesion molecules, IV- Matrix metalloproteases, and V-Others. Significant differences between the measured concentrations were tested using Mann-Whitney U test (MW). **(B and D)** Random forest results shown as the mean decrease Gini for each variable. SOFA: Sequential organ failure assessment score, SS: Septic shock, Amp,: amputation, Type: microbiological classification of NSTI.

Table S3. Results from the ROC analyses discriminating septic shock and non-septic shock presence in all cases, type I, and type II NSTI in the discovery cohort. The results are shown as the mean values of the iterations. Thresholds are given as pg/ml. (*Continues in the next page*)

	All					Type I					Type II				
Analyte	AUC	[95% CI]	Threshold	Sn	Sp	AUC	[95% CI]	Threshold	Sn	Sp	AUC	[95% CI]	Threshold	Sn	Sp
CCL2/MCP-1	0.75	[0.68-0.82]	620.8	0.69	0.76	0.69	[0.58-0.8]	557.19	0.64	0.71	0.79	[0.7-0.88]	625.11	0.77	0.78
CCL4/MIP-1β	0.72	[0.65-0.79]	793.25	0.75	0.68	0.66	[0.55-0.77]	793.06	0.67	0.72	0.78	[0.68-0.87]	827.33	0.71	0.74
CCL5/RANTES	0.53	[0.46-0.61]	7656.11	0.58	0.5	0.51	[0.4-0.63]	7155.43	0.56	0.51	0.56	[0.44-0.67]	6179.53	0.55	0.6
CXCL-8/IL-8	0.75	[0.69-0.82]	38.95	0.72	0.71	0.76	[0.67-0.86]	47.59	0.66	0.76	0.75	[0.65-0.85]	36.18	0.74	0.74
CXCL10/IP-10	0.67	[0.6-0.74]	324.19	0.59	0.67	0.51	[0.39-0.62]	103.24	0.49	0.62	0.7	[0.6-0.8]	1297.83	0.64	0.67
IL-1α	0.75	[0.69-0.82]	49.05	0.67	0.78	0.72	[0.61-0.82]	46.28	0.63	0.76	0.77	[0.68-0.86]	51.51	0.73	0.77
IL-1β	0.69	[0.62-0.76]	15.94	0.63	0.68	0.67	[0.56-0.77]	18.19	0.57	0.74	0.72	[0.62-0.82]	15.16	0.69	0.7
IL-2	0.72	[0.65-0.79]	498.43	0.66	0.69	0.66	[0.55-0.77]	371.54	0.61	0.7	0.75	[0.65-0.84]	663.96	0.73	0.66
IL-4	0.76	[0.69-0.82]	201.45	0.69	0.77	0.69	[0.58-0.8]	178.91	0.68	0.68	0.79	[0.7-0.89]	206.75	0.79	0.76
IL-6	0.78	[0.71-0.84]	419.48	0.71	0.78	0.74	[0.64-0.84]	402.75	0.66	0.81	0.79	[0.71-0.88]	608.89	0.72	0.79
IL-10	0.75	[0.68-0.81]	32.1	0.71	0.7	0.7	[0.6-0.8]	22.01	0.6	0.76	0.77	[0.68-0.86]	60.92	0.67	0.78
IL-12p70	0.72	[0.65-0.79]	154.45	0.65	0.8	0.68	[0.57-0.79]	138.16	0.6	0.77	0.74	[0.64-0.84]	155.7	0.73	0.75
IL-13	0.72	[0.65-0.79]	1276.59	0.67	0.71	0.7	[0.59-0.8]	1274.32	0.61	0.77	0.72	[0.61-0.82]	1306.58	0.68	0.68
IL-17A	0.69	[0.62-0.76]	19.92	0.62	0.69	0.64	[0.53-0.75]	13.14	0.73	0.61	0.72	[0.62-0.83]	23.6	0.69	0.73
IL-18	0.63	[0.55-0.7]	458.2	0.59	0.59	0.6	[0.48-0.71]	457.81	0.51	0.69	0.61	[0.5-0.72]	455.79	0.69	0.51
IL-22	0.72	[0.66-0.79]	98.66	0.7	0.72	0.67	[0.56-0.78]	94.12	0.59	0.75	0.74	[0.64-0.84]	109.44	0.73	0.69
IL-36β/IL-1F8	0.74	[0.68-0.81]	15.91	0.64	0.8	0.67	[0.56-0.78]	13.74	0.66	0.65	0.8	[0.71-0.89]	16.25	0.74	0.8
E-Selectin	0.64	[0.56-0.71]	106330.13	0.61	0.64	0.58	[0.47-0.69]	82682.14	0.58	0.58	0.65	[0.54-0.76]	127423.41	0.66	0.66
ICAM-1	0.63	[0.56-0.71]	590697.9	0.6	0.66	0.59	[0.48-0.71]	545439.14	0.6	0.65	0.66	[0.55-0.76]	663488.8	0.58	0.71
VCAM-1	0.61	[0.54-0.69]	3820864	0.58	0.61	0.49	[0.37-0.6]	2786263.5	0.58	0.51	0.65	[0.54-0.76]	4147485	0.64	0.65
MMP-1	0.61	[0.54-0.69]	1887.25	0.53	0.64	0.63	[0.51-0.74]	2132.29	0.54	0.66	0.63	[0.52-0.74]	1545.95	0.62	0.62
MMP-8	0.71	[0.64-0.78]	26460.02	0.72	0.65	0.72	[0.61-0.82]	32722.57	0.66	0.73	0.71	[0.61-0.82]	23445.25	0.73	0.69
MMP-9	0.7	[0.63-0.77]	12544.29	0.69	0.67	0.64	[0.54-0.75]	17731.22	0.64	0.64	0.72	[0.62-0.82]	12229.85	0.82	0.6
C5/C5a	0.52	[0.44-0.6]	19591.17	0.51	0.56	0.51	[0.39-0.62]	22388.13	0.6	0.5	0.54	[0.43-0.66]	19283.92	0.51	0.63
Collagen-IVα1	0.75	[0.68-0.81]	1933.49	0.71	0.71	0.72	[0.61-0.82]	1851.14	0.64	0.75	0.76	[0.66-0.85]	2078.03	0.76	0.67
Fas-Ligand	0.66	[0.58-0.73]	26.45	0.6	0.65	0.52	[0.41-0.64]	20.43	0.57	0.58	0.74	[0.64-0.84]	31.58	0.72	0.66
Galectin-3	0.64	[0.57-0.72]	3283.99	0.6	0.64	0.69	[0.59-0.8]	3625.1	0.59	0.75	0.61	[0.5-0.72]	3230.36	0.61	0.63
G-CSF	0.79	[0.73-0.85]	673.45	0.76	0.76	0.77	[0.67-0.86]	501.72	0.7	0.8	0.81	[0.73-0.9]	1487.47	0.75	0.84
I-alpha-1/COL1A1	0.68	[0.6-0.75]	7805.59	0.66	0.63	0.64	[0.53-0.75]	7428.67	0.56	0.72	0.67	[0.56-0.78]	8715.61	0.66	0.61
MPO	0.68	[0.61-0.75]	40200.88	0.58	0.74	0.63	[0.52-0.74]	43515.31	0.53	0.76	0.73	[0.63-0.83]	36569.84	0.7	0.72
Pentraxin-3	0.74	[0.68-0.81]	7975.61	0.76	0.68	0.69	[0.58-0.79]	7317.16	0.68	0.68	0.77	[0.68-0.87]	9456.21	0.81	0.71
Resistin	0.63	[0.55-0.7]	50956.18	0.65	0.61	0.62	[0.5-0.73]	52193.54	0.63	0.66	0.63	[0.52-0.74]	49776.07	0.69	0.59
S100A8	0.78	[0.71-0.84]	819.42	0.66	0.83	0.75	[0.65-0.84]	661.36	0.69	0.74	0.79	[0.7-0.89]	872.17	0.74	0.84

S100A9	0.55	[0.48-0.63]	3343.11	0.5	0.62	0.55	[0.44-0.67]	4018.29	0.54	0.6	0.58	[0.47-0.69]	2716.59	0.52	0.64
Thrombomodulin	0.62	[0.54-0.69]	12989.07	0.52	0.71	0.54	[0.43-0.66]	13253.96	0.45	0.7	0.68	[0.58-0.79]	12907.4	0.57	0.78
TNFα	0.72	[0.65-0.79]	26.3	0.67	0.71	0.64	[0.53-0.75]	25.64	0.56	0.77	0.77	[0.68-0.86]	30.2	0.71	0.74
WBC	0.60	[0.52-0.68]	14.72	0.55	0.66	0.61	[0.5-0.73]	15.50	0.55	0.69	0.58	[0.47-0.7]	14.02	0.58	0.62
CRP	0.51	[0.43-0.59]	322.79	0.46	0.64	0.57	[0.45-0.68]	383.58	0.47	0.77	0.52	[0.4-0.64]	241.02	0.49	0.64
Creatinine	0.63	[0.56-0.71]	131.10	0.61	0.65	0.55	[0.43-0.66]	115.03	0.55	0.60	0.70	[0.59-0.81]	140.79	0.69	0.68

Type I: Polymicrobial culture, Type II: monomicrobial culture, AUC: Area under the curve, CI: Confidence interval, Sn: Sensitivity, Sp: Specificity, WBC: White blood cells, CRP: C-reactive protein.

Table S4. Results of the random forest models discriminating septic shock and non- septic shock presence in all cases, type I, and type II NSTI in the discovery cohort. The p-values of the random forest results were calculated using 100 permutations of the original dataset. (*Continues in the next page*)

Analyte	All								Type I								Type II							
	Only proteins				with Clinical variables				Only proteins				with Clinical variables				Only proteins				with Clinical variables			
	MDA	p-val	MDG	p-val	MDA	p-val	MDG	p-val	MDA	p-val	MDG	p-val	MDA	p-val	MDG	p-val	MDA	p-val	MDG	p-val	MDA	p-val	MDG	p-val
CCL2/MCP-1	97	**0.01**	5.2	**0.01**	85	**0.01**	3.7	**0.03**	10	0.31	1.4	0.74	12	0.12	1.0	0.73	73	**0.01**	3.0	**0.01**	82	**0.01**	2.7	**0.01**
CCL4/MIP-1β	70	**0.02**	3.6	0.28	79	**0.01**	3.2	0.06	43	**0.05**	1.5	0.45	62	**0.01**	1.7	0.06	52	**0.02**	1.9	0.10	51	**0.01**	1.6	0.06
CCL5/RANTES	-15	0.73	2.3	1.00	-21	0.89	1.5	1.00	-25	0.86	0.8	1.00	-25	0.85	0.6	1.00	13	0.16	1.3	1.00	-3	0.33	0.8	1.00
CXCL-8/IL-8	157	**0.01**	5.4	**0.01**	146	**0.01**	5.5	**0.01**	101	**0.01**	3.4	**0.02**	103	**0.01**	3.0	**0.01**	102	**0.01**	2.5	0.09	104	**0.01**	2.5	**0.05**
CXCL10/IP-10	51	**0.01**	2.4	1.00	7	0.30	1.3	1.00	5	0.34	1.0	1.00	-4	0.39	0.7	1.00	65	**0.01**	1.3	0.93	36	**0.03**	0.8	1.00
IL-1α	95	**0.01**	4.2	0.08	79	**0.01**	3.6	**0.04**	68	**0.02**	2.1	0.11	43	**0.03**	1.6	0.12	50	**0.02**	1.6	0.33	57	**0.02**	1.5	0.18
IL-1β	15	0.17	2.6	1.00	27	0.07	1.8	1.00	9	0.17	1.2	0.85	9	0.21	0.9	0.96	3	0.39	1.1	1.00	17	0.19	0.9	0.99
IL-2	44	**0.03**	2.7	1.00	45	**0.02**	2.2	0.88	32	0.09	1.4	0.56	39	**0.02**	1.4	0.19	30	0.08	1.1	0.99	32	0.06	1.0	0.89
IL-4	100	**0.02**	4.8	**0.02**	97	**0.01**	4.0	**0.02**	40	**0.04**	1.5	0.26	38	**0.04**	1.4	0.09	99	**0.01**	3.1	**0.01**	98	**0.01**	2.7	**0.01**
IL-6	144	**0.01**	8.8	**0.01**	123	**0.01**	6.2	**0.01**	86	**0.01**	3.5	**0.01**	111	**0.01**	3.3	**0.01**	98	**0.01**	3.0	**0.01**	77	**0.02**	1.9	**0.05**
IL-10	95	**0.01**	4.1	0.24	64	**0.02**	2.6	0.45	28	0.11	1.3	0.86	19	0.16	1.0	0.87	74	**0.01**	1.8	0.36	58	**0.01**	1.5	0.24
IL-12p70	61	**0.01**	4.0	0.08	33	**0.02**	2.7	0.15	41	**0.02**	1.7	0.11	17	0.07	1.2	0.28	16	0.19	1.4	0.79	20	0.14	1.0	0.67
IL-13	23	**0.05**	2.0	1.00	26	**0.05**	1.8	0.99	69	**0.02**	2.0	**0.05**	49	**0.01**	1.7	**0.05**	11	0.21	0.9	1.00	9	0.29	0.6	1.00
IL-17A	45	**0.04**	2.2	1.00	51	**0.02**	1.8	1.00	16	0.14	1.0	1.00	17	0.12	0.8	0.99	22	0.11	1.2	0.99	72	**0.01**	1.6	0.25
IL-18	49	**0.03**	2.6	1.00	53	**0.03**	1.9	1.00	-2	0.47	0.9	1.00	6	0.20	0.8	1.00	50	**0.02**	1.6	0.90	48	0.07	1.1	0.97
IL-22	53	**0.02**	3.8	0.11	38	**0.02**	2.4	0.51	40	**0.05**	2.0	0.12	33	**0.04**	1.7	0.07	41	**0.04**	1.2	0.99	34	**0.05**	0.9	0.99
IL-36β/IL-1F8	95	**0.01**	4.6	**0.01**	69	**0.01**	3.3	**0.01**	47	**0.02**	1.3	0.64	25	0.07	1.0	0.53	93	**0.01**	3.1	**0.01**	69	**0.02**	1.9	**0.03**
E-Selectin	1	0.41	2.1	1.00	4	0.37	1.6	1.00	3	0.28	0.8	1.00	-9	0.48	0.6	1.00	7	0.31	1.2	1.00	14	0.19	1.0	0.97
ICAM-1	36	0.06	2.3	1.00	24	0.11	1.5	1.00	5	0.29	1.1	1.00	4	0.36	0.8	1.00	8	0.31	0.8	1.00	1	0.45	0.7	1.00
VCAM-1	-7	0.52	2.1	1.00	-10	0.55	1.4	1.00	3	0.30	1.0	1.00	9	0.27	0.7	1.00	2	0.37	1.1	1.00	3	0.30	0.7	1.00
MMP-1	48	**0.03**	2.5	1.00	8	0.25	1.5	1.00	48	**0.05**	1.8	0.38	19	0.15	1.0	0.88	-18	0.83	1.0	1.00	-22	0.94	0.8	1.00
MMP-8	87	**0.01**	3.3	0.83	79	**0.01**	2.7	0.49	85	**0.01**	2.6	**0.03**	71	**0.01**	1.9	**0.02**	27	0.09	1.3	0.98	37	**0.04**	0.9	1.00
MMP-9	60	**0.01**	3.6	0.67	50	**0.02**	2.2	0.96	8	0.26	1.4	0.83	5	0.30	1.0	0.96	75	**0.03**	1.8	0.49	66	**0.04**	1.4	0.46
C5/C5a	9	0.25	3.2	0.99	-1	0.41	2.1	0.99	-21	0.82	1.0	1.00	-18	0.75	0.7	1.00	30	0.11	1.9	0.55	12	0.23	1.1	0.99
Collagen-IVα1	63	**0.01**	3.3	0.86	42	**0.04**	1.9	1.00	39	0.06	1.5	0.49	35	**0.05**	1.2	0.54	31	**0.04**	1.3	1.00	11	0.26	0.7	1.00
Fas-Ligand	24	**0.04**	2.4	1.00	30	0.10	1.6	1.00	-1	0.41	1.0	1.00	2	0.30	0.8	1.00	80	**0.02**	1.7	0.65	73	**0.03**	1.4	0.43
Galectin-3	43	**0.04**	2.6	1.00	63	**0.02**	2.3	0.94	41	0.07	1.5	0.59	44	**0.04**	1.5	0.27	0	0.38	0.8	1.00	27	0.11	0.8	1.00
G-CSF	196	**0.01**	11.1	**0.01**	150	**0.01**	6.5	**0.01**	133	**0.01**	4.2	**0.01**	112	**0.01**	2.8	**0.01**	126	**0.01**	4.5	**0.01**	125	**0.01**	3.7	**0.01**
I-alpha-1/COL1A1	21	0.07	2.8	1.00	34	0.08	2.1	0.98	30	0.10	1.7	0.49	24	0.07	1.2	0.72	-9	0.48	1.1	1.00	-4	0.44	0.8	1.00
MPO	92	**0.01**	3.2	0.96	69	**0.01**	2.3	0.92	12	0.25	1.3	0.87	22	0.11	1.1	0.66	74	**0.01**	1.9	0.36	50	**0.03**	1.1	0.91
Pentraxin-3	117	**0.01**	6.1	**0.01**	81	**0.02**	3.3	0.13	30	0.11	1.5	0.59	9	0.17	0.9	0.97	93	**0.02**	3.3	**0.03**	104	**0.01**	2.8	**0.02**
Resistin	23	0.10	2.4	1.00	-1	0.43	1.6	1.00	19	0.18	1.6	0.59	2	0.34	1.1	0.75	-28	0.93	0.7	1.00	-14	0.68	0.5	1.00

S100A8	160	**0.01**	7.7	**0.01**	162	**0.01**	7.2	**0.01**	115	**0.01**	3.2	**0.01**	92	**0.01**	2.4	**0.02**	108	**0.01**	3.9	**0.01**	123	**0.01**	3.7	**0.01**
S100A9	19	0.13	2.3	1.00	14	0.24	1.8	1.00	13	0.15	1.0	1.00	6	0.29	0.8	1.00	19	0.12	1.1	1.00	15	0.13	0.8	1.00
Thrombomodulin	53	**0.02**	3.1	1.00	29	**0.02**	2.5	0.82	-2	0.48	1.3	0.94	-1	0.33	1.1	0.87	44	**0.03**	2.0	0.36	31	0.06	1.3	0.67
TNFα	72	**0.02**	3.5	0.50	62	**0.02**	3.0	0.18	4	0.21	1.2	0.72	10	0.13	1.0	0.72	62	**0.04**	2.0	0.17	66	**0.01**	2.0	**0.04**
Age	NA	NA	NA	NA	-1	0.37	1.7	1.00	NA	NA	NA	NA	-4	0.50	0.9	0.95	NA	NA	NA	NA	25	0.08	1.0	0.96
Sex	NA	NA	NA	NA	22	**0.03**	0.4	0.13	NA	NA	NA	NA	41	**0.01**	0.6	**0.01**	NA	NA	NA	NA	0	0.26	0.1	0.96
SOFA	NA	NA	NA	NA	165	**0.01**	4.2	**0.01**	NA	NA	NA	NA	75	**0.01**	1.5	**0.04**	NA	NA	NA	NA	117	**0.01**	2.0	**0.02**
WBC	NA	NA	NA	NA	15	0.23	1.9	1.00	NA	NA	NA	NA	-4	0.36	1.0	0.94	NA	NA	NA	NA	10	0.26	0.8	1.00
CRP	NA	NA	NA	NA	24	0.08	1.9	1.00	NA	NA	NA	NA	11	0.19	1.0	0.92	NA	NA	NA	NA	22	0.13	1.0	1.00
Creatinine	NA	NA	NA	NA	23	0.14	2.0	1.00	NA	NA	NA	NA	14	0.16	1.1	0.81	NA	NA	NA	NA	21	0.09	0.9	1.00

AUC: Area under the curve, CI: Confidence interval, Sn: Sensitivity, Sp: Specificity, MDA: Mean decrease accuracy, MDG: Mean decrease gini, SOFA: Sequential organ failure assessment score, SS: Septic shock, WBC: White blood cells, CRP: C-reactive protein.

Table S5. Clinical characteristics of the validation patient cohort and the additional sepsis cohort. The data are shown as mean values and standard deviations, or percentages.

	NSTI				p-value[C]	
	All (60)	Type I (39)	Type II (21)	Sepsis (24)	NSTI vs Sepsis	Type I vs Type II
Age (years)	58 ± 14	58 ± 11	57 ± 18	69 ± 11	0.001	0.803
Sex (male)	39 (65%)	24 (62%)	15 (71%)	16 (67%)	>0.999	0.573
Septic shock at baseline	29 (48%)	17 (44%)	12 (57%)	11 (46%)	>0.999	0.506
Amputation[A]	3 (14%)	1 (17%)	2 (13%)	NA	NA	>0.999
90-day mortality	8 (13%)	5 (13%)	3 (14%)	4 (17%)	0.735	>0.999
Comorbidities	40 (67%)	26 (67%)	14 (67%)	NA	NA	>0.999
Diabetes (Type I or II)	13 (48%)	11 (61%)	2 (22%)	NA	NA	0.114
Cardiovascular disease	27 (45%)	18 (46%)	9 (43%)	NA	NA	>0.999
Surgery Before NSTI[B]	16 (27%)	14 (36%)	2 (10%)	NA	NA	0.034
SAPS II	44 ± 15 (3% NA)	42 ± 13 (3% NA)	49 ± 17 (5% NA)	NA	NA	0.090
SOFA at admission	8 ± 3 (2% NA)	7 ± 3 (0% NA)	8 ± 4 (5% NA)	4 ± 2 (0% NA)	<0.0001	0.425
Type I	39 (65%)	NA	NA	NA	NA	NA
Microbiological findings						
GAS	8 (13%)	1 (3%)	7 (33%)	2 (8%)	0.717	0.002
Other Strep	3 (5%)	2 (5%)	1 (5%)	0	0.554	>0.999
S. aureus	7 (12%)	2 (5%)	5 (24%)	2 (8%)	>0.999	0.045
Clostridium sp.	1 (2%)	1 (3%)	0 (0%)	0	>0.999	>0.999
Others	41 (68%)	33 (85%)	8 (38%)	20 (83%)	0.188	<0.001

NSTI: Necrotizing soft tissue infection, type I: Polymicrobial culture, type II: monomicrobial etiology, NA: not applicable, SAPS II: Simplified acute physiology score II, SOFA: Sequential organ failure assessment score, GAS: Group A *Streptococcus*, Strep: *Streptococcus sp.*

[A] Includes only infections in extremities (N=22; Type I=6)

[B] Within 4 weeks before admission for NSTI

[C] Significant differences between cohorts were determined by Mann-Whitney U test or Fisher's exact test.

Table S6. Statistical comparison of the characteristics of discovery cohort compared to the validation cohort.

	p-value[C]		
	Discovery vs verification		
	All	Type I	Type II
Age (years)	0.445	0.576	0.546
Sex (male)	0.192	0.852	0.103
Septic shock at baseline	0.565	0.853	>0.999
Amputation[A]	0.435	>0.999	>0.999
90-day mortality	0.353	0.252	>0.999
Comorbidities	0.434	0.211	>0.999
Diabetes (Type I or II)	0.866	0.437	>0.999
Cardiovascular disease	0.560	0.581	0.813
Surgery Before NSTI[B]	0.035	0.142	0.667
SAPS II	0.849	0.465	0.366
SOFA at admission	0.141	0.534	0.412
Type I	0.014	NA	NA
Microbiological findings			
GAS	0.0001	0.294	0.007
Other Strep	0.035	0.067	0.314
S. aureus	0.278	0.731	0.012
Clostridium sp.	0.475	0.681	>0.999
Others	<0.0001	0.006	0.003

NSTI: Necrotizing soft tissue infection, Type I: Polymicrobial culture, type II: monomicrobial etiology, NA: not applicable, SAPS II: Simplified acute physiology score II, SOFA: Sequential organ failure assessment score, GAS: Group A *Streptococcus*, Strep: *Streptococcus sp.*

[A] Includes only infections in extremities

[B] Within 4 weeks before admission for NSTI

[C] Significant differences between cohorts were determined by Mann-Whitney U test or Fisher's exact test.

Table S7. Results of the ROC analyses applied in the validation cohort. Thresholds are given in pg/ml.

	Comparison	Analyte	AUC	[95% CI]	Threshold	Sensitivity	Specificity
NSTI	Sepsis vs NSTI	Thrombomodulin	0.77	[0.67-0.88]	3972.01	0.75	0.75
		WBC	0.75	[0.63-0.87]	13.30	0.76	0.79
		CRP	0.75	[0.63-0.87]	153	0.83	0.67
		Creatinine	0.52	[0.39-0.66]	114.50	0.61	0.58
Type	NSTI cohort Type I vs Type II	CXCL-10	0.78	[0.65-0.91]	36.43	0.71	0.74
		MMP-9	0.66	[0.51-0.81]	34902.50	0.71	0.54
		IL-10	0.63	[0.47-0.79]	43.5	0.52	0.74
		Fas-Ligand	0.55	[0.39-0.72]	1.88	0.79	0.42
		IL-2	0.64	[0.49-0.78]	6.02	0.71	0.53
		WBC	0.57	[0.4-0.74]	14.30	0.48	0.79
		CRP	0.50	[0.34-0.66]	242.5	0.52	0.61
		Creatinine	0.61	[0.44-0.77]	106.00	0.62	0.63
	NSTI cohort Type I vs GAS	CXCL-10	0.99	[0.97-1]	642.77	1	0.92
		MMP-9	0.60	[0.33-0.87]	16960.00	0.43	0.90
		IL-10	0.79	[0.6-0.99]	53.41	0.71	0.77
		Fas-Ligand	0.74	[0.56-0.92]	2.61	0.86	0.58
		IL-2	0.82	[0.65-0.99]	8.01	0.71	0.82
		WBC	0.58	[0.28-0.87]	21.20	0.57	0.66
		CRP	0.51	[0.25-0.77]	286	0.57	0.53
		Creatinine	0.70	[0.45-0.95]	150	0.71	0.76
Septic Shock	NSTI cohort no SS vs SS	G-CSF	0.76	[0.64-0.88]	334.3	0.55	0.97
		S100A8	0.72	[0.58-0.85]	289.47	0.57	0.89
		IL-6	0.82	[0.71-0.93]	467.5	0.62	0.97
		WBC	0.51	[0.36-0.67]	20.8	0.69	0.47
		CRP	0.64	[0.5-0.78]	265.5	0.62	0.63
		Creatinine	0.69	[0.56-0.83]	97.5	0.76	0.67
	Sepsis cohort no SS vs SS	G-CSF	0.83	[0.65-1]	23.66	0.73	0.83
		S100A8	0.76	[0.57-0.96]	112.96	0.73	0.69
		IL-6	0.85	[0.68-1]	308.81	0.8	0.77
		WBC	0.47	[0.21-0.74]	10.65	0.64	0.62
		CRP	0.44	[0.19-0.69]	105	0.64	0.54
		Creatinine	0.93	[0.79-1]	117.5	1	0.92

NSTI: Necrotizing soft tissue infection, type I: Polymicrobial culture, type II: monomicrobial culture, GAS: Group A *Streptococcus*, SS: Septic shock, CI: Confidence interval, WBC: White blood cells, CRP: C-reactive protein

Table S8. Uni- and multivariate logistic regression analyses performed in the validation cohort.

			Univariate			Multivariate*		
	Samples	Analyte	OR	[95% CI]	p-value	OR	[95% CI]	p-value
NSTI	Sepsis vs NSTI	Thrombomodulin	2.79	[1.63-5.18]	<0.001	3.01	[1.42-7.52]	0.003
		WBC	2.29	[1.41-4.02]	<0.001	2.62	[1.34-5.93]	0.00
		CRP	2.31	[1.48-3.85]	<0.001	1.85	[1.03-3.5]	0.039
		Creatinine	0.95	[0.54-1.71]	0.86	0.58	[0.23-1.33]	0.20
Type	NSTI cohort<br>Type I vs Type II	CXCL-10	1.37	[1.15-1.7]	<0.001	1.39	[1.16-1.76]	<0.001
		MMP-9	0.60	[0.36-0.95]	0.03	0.49	[0.27-0.82]	0.01
		IL-10	1.23	[1-1.54]	0.045	1.31	[1.04-1.71]	0.023
		Fas-Ligand	1.15	[0.69-1.94]	0.59	1.06	[0.63-1.8]	0.84
		IL-2	1.28	[0.78-2.22]	0.321	1.22	[0.72-2.17]	0.45
		WBC	0.66	[0.35-1.2]	0.17	0.63	[0.32-1.15]	0.14
		CRP	1.06	[0.6-1.92]	0.849	1.05	[0.59-1.91]	0.87
		Creatinine	1.65	[0.9-3.19]	0.11	1.64	[0.86-3.32]	0.14
	NSTI cohort<br>Type I vs GAS	CXCL-10	2.12	[1.43-21.4]	<0.001	1.68	[1.3-5.23]	<0.001
		MMP-9	0.74	[0.35-1.49]	0.39	0.69	[0.31-1.4]	0.31
		IL-10	1.52	[1.14-2.25]	0.003	1.92	[1.23-3.53]	0.003
		Fas-Ligand	2.16	[0.97-6.26]	0.06	2.64	[1.03-9.94]	0.04
		IL-2	1.78	[1.03-3.33]	0.041	1.96	[0.96-4.73]	0.064
		WBC	0.89	[0.39-2.58]	0.80	0.93	[0.42-2.44]	0.87
		CRP	0.96	[0.45-2.38]	0.928	0.88	[0.39-2.16]	0.768
		Creatinine	2.25	[0.93-6]	0.072	2	[0.73-6.48]	0.181
Septic Shock	NSTI cohort<br>no SS vs SS	G-CSF	1.33	[1.13-1.67]	<0.001	1.37	[1.14-1.76]	<0.001
		S100A8	1.3	[1.07-1.61]	0.006	1.25	[1.02-1.59]	0.028
		IL-6	1.61	[1.28-2.18]	<0.001	1.51	[1.19-2.03]	<0.001
		WBC	0.73	[0.38-1.3]	0.286	0.53	[0.18-1.17]	0.125
		CRP	1.68	[0.96-3.2]	0.072	1.48	[0.76-3.06]	0.254
		Creatinine	2.06	[1.1-4.26]	0.023	1.47	[0.71-3.17]	0.304
	Sepsis cohort<br>no SS vs SS	G-CSF	1.39	[1.11-2.37]	0.002	1.36	[1.03-2.52]	0.028
		S100A8	1.54	[1.05-2.62]	0.027	1.27	[0.71-2.64]	0.431
		IL-6	1.41	[1.12-2.15]	0.001	1.3	[1-1.98]	0.046
		WBC	0.72	[0.33-1.35]	0.308	0.65	[0.23-1.43]	0.286
		CRP	0.79	[0.41-1.43]	0.429	0.54	[0.13-1.48]	0.244
		Creatinine	17.7	[3.03-331.92]	<0.001	21.43	[1.9-6.5E12]	0.006

NSTI: Necrotizing soft tissue infection, Type I: Polymicrobial culture, Type I: monomicrobial culture, GAS: Group A *Streptococcus*, SS: Septic shock, CI: Confidence interval, WBC: White blood cells, CRP: C-reactive protein.

*Logistic regression including age, sex and SOFA

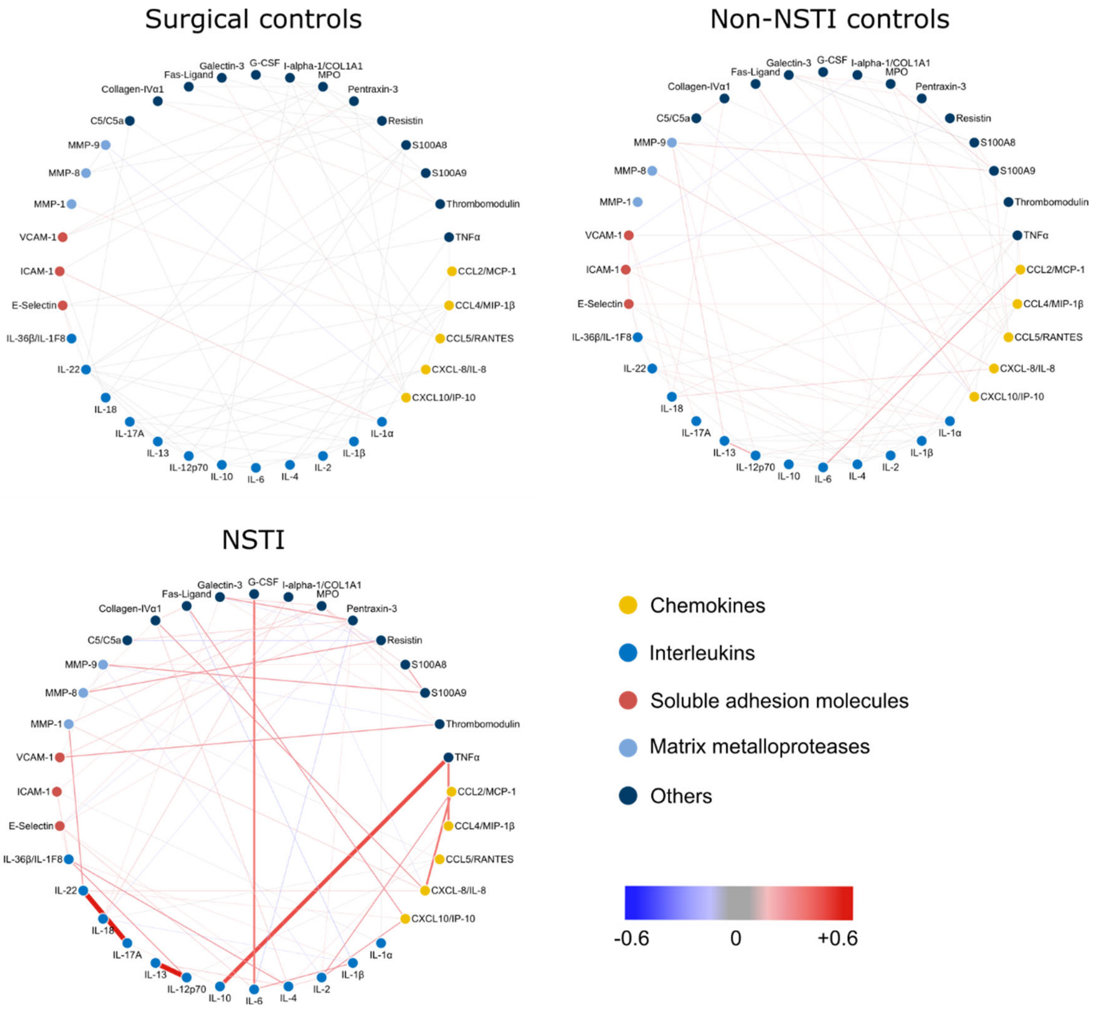

Figure S4. Association networks of measured analytes in the surgical control, Non-NSTI control and NSTI discovery cohorts. The non- NSTI patients are suspected NSTI cases but that had no necrotic tissue upon surgical exploration. The strength of the partial correlation between analytes is shown by the color and the weight of the connection.

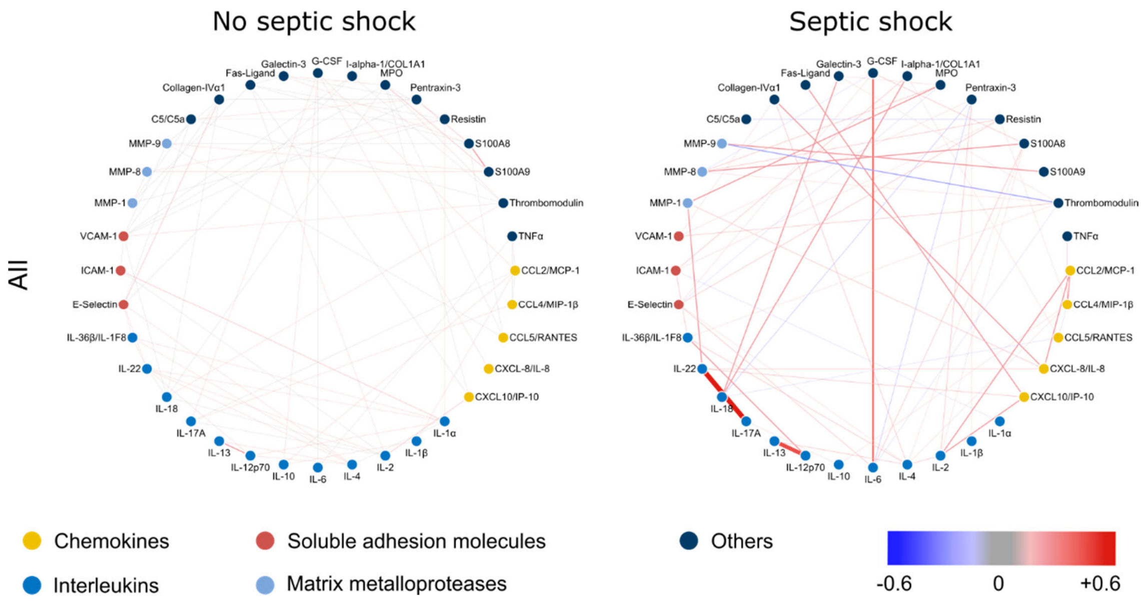

Figure S5. Association networks of measured analytes in samples of NSTI patients with or without septic shock. The networks are based on the discovery cohort measurements. The colors of the analytes indicate the category of the analytes. The strength of the partial correlation between analytes is shown by the color and the weight of the connection.

Table S9. Connectivity values for each measured analyte in the discovery cohort. Samples were aggregated by type. The differential connectivity and q-values were calculated for the comparison of septic shock development within each NSTI type. (*Continues in the next page*)

		Type I				Type II			
		Connectivity		Differential connectivity	q-value	Connectivity		Differential connectivity	q-value
	Analyte	no SS	SS			no SS	SS		
I	CCL2/MCP-1	0.74	0.49	0.26	0.002	0.50	0.77	0.27	0.001
	CCL4/MIP-1β	0.21	0.34	0.13	0.003	0.28	0.55	0.26	0.001
	CCL5/RANTES	0.49	0.33	0.16	0.010	0.37	0.77	0.39	0.001
	CXCL-8/IL-8	0.70	0.30	0.41	0.002	0.48	0.54	0.06	0.041
	CXCL10/IP-10	0.26	0.47	0.21	0.002	0.32	0.87	0.55	0.001
II	IL-1α	**0.81**	**0.17**	**0.64**	**0.002**	0.69	1.07	0.38	0.001
	IL-1β	0.70	0.29	0.40	0.002	0.46	0.51	0.05	0.097
	IL-2	0.62	0.66	0.03	0.313	0.50	0.87	0.37	0.001
	IL-4	**0.90**	**0.29**	**0.61**	**0.002**	0.61	0.63	0.02	0.505
	IL-6	0.67	0.27	0.39	0.002	0.31	0.68	0.36	0.001
	IL-10	0.00	0.00	0.00	0.833	0.37	0.11	0.26	0.001
	IL-12p70	0.81	0.98	0.17	0.003	0.59	0.68	0.08	0.034
	IL-13	0.49	0.75	0.25	0.002	0.59	0.86	0.27	0.001
	IL-17A	**0.09**	**0.73**	**0.64**	**0.002**	0.30	0.59	0.28	0.001
	IL-18	0.52	0.13	0.39	0.002	0.30	0.90	0.60	0.001
	IL-22	0.69	0.72	0.02	0.454	0.64	0.85	0.21	0.001
	IL-36β/IL-1F8	0.96	0.85	0.12	0.025	0.42	0.65	0.23	0.001
III	E-Selectin	0.74	0.88	0.14	0.018	0.55	0.87	0.32	0.001
	ICAM-1	0.27	0.55	0.28	0.002	0.53	0.84	0.31	0.001
	VCAM-1	0.84	0.36	0.48	0.002	0.29	0.29	0.00	0.767
IV	MMP-1	0.54	0.37	0.17	0.003	0.18	0.89	0.71	0.001
	MMP-8	0.55	0.37	0.18	0.002	0.40	0.69	0.28	0.001
	MMP-9	0.20	0.58	0.38	0.002	0.46	0.14	0.31	0.001
V	C5/C5a	0.47	0.13	0.34	0.002	0.31	0.64	0.33	0.001
	Collagen-IVα1	0.51	0.64	0.13	0.010	0.21	0.59	0.38	0.001
	Fas-Ligand	0.21	0.44	0.23	0.002	0.24	0.38	0.14	0.011
	Galectin-3	0.40	0.28	0.13	0.009	**0.34**	**1.19**	**0.84**	**0.001**
	G-CSF	0.68	0.20	0.48	0.002	0.41	1.15	0.74	0.001
	I-alpha-1/COL1A1	0.40	0.44	0.04	0.205	**0.28**	**1.03**	**0.74**	**0.001**
	MPO	0.53	0.42	0.10	0.049	0.83	0.71	0.12	0.006
	Pentraxin-3	0.45	0.48	0.03	0.293	0.27	0.93	0.66	0.001
	Resistin	0.97	1.01	0.04	0.313	0.26	0.89	0.63	0.001
	S100A8	0.29	0.33	0.04	0.205	0.59	0.84	0.25	0.001
	S100A9	0.59	0.42	0.17	0.002	0.53	0.49	0.05	0.075

Thrombomodulin	0.42	0.46	0.04	0.313	**0.49**	**1.46**	**0.98**	**0.001**
TNFα	0.82	0.40	0.42	0.002	0.57	0.74	0.18	0.001

Categories: I-Chemokines, II-Interleukins, III-Soluble adhesion molecules, IV- Matrix metalloproteases, and V-Others. Type I: Polymicrobial culture, SS: Septic shock.

Table S10. Summary of the raw data for the discovery cohort. The numbers of values below (OOR <) or over (OOR >) the range are displayed in the first set of columns. The last two sets of columns display the statistical summary of the data before and after imputation. (*Continues in the next page*)

	Analyte	OOR data						*Raw*			*Imputed*		
		OOR >		OOR <		Total		Median pg/ml	Mean pg/ml	SD	Median pg/ml	Mean pg/ml	SD
		#	%	#	%	#	%						
I	CCL2/MCP-1	0	0%	0	0%	0	0%	438	1840	5570	438	1840	5570
	CCL4/MIP-1β	0	0%	0	0%	0	0%	768	811	453	768	811	453
	CCL5/RANTES	10	3%	0	0%	10	3%	6523	10268	10954	6753	11893	13979
	CXCL-8/IL-8	5	2%	1	0%	6	2%	30	321	1408	31	563	2358
	CXCL10/IP-10	40	13%	0	0%	40	13%	151	1730	5981	197	51320	128964
II	IL-1α	0	0%	0	0%	0	0%	44	58	228	44	58	228
	IL-1β	0	0%	49	16%	49	16%	16	31	62	13	26	58
	IL-2	0	0%	18	6%	18	6%	473	676	577	426	637	581
	IL-4	0	0%	1	0%	1	0%	180	187	71	180	186	72
	IL-6	20	6%	0	0%	20	6%	225	2056	7647	263	6372	18022
	IL-10	1	0%	24	8%	25	8%	31	151	672	25	168	816
	IL-12p70	0	0%	44	14%	44	14%	141	168	179	128	144	176
	IL-13	0	0%	0	0%	0	0%	1159	1220	653	1159	1220	653
	IL-17A	0	0%	36	12%	36	12%	17	48	144	15	42	136
	IL-18	2	1%	0	0%	2	1%	416	699	1092	423	760	1327
	IL-22	0	0%	5	2%	5	2%	94	104	133	93	102	132
	IL-36β/IL-1F8	0	0%	0	0%	0	0%	14	14	6	14	14	6
III	E-Selectin	1	0%	0	0%	1	0%	88779	111397	104435	89170	114977	121780
	ICAM-1	0	0%	0	0%	0	0%	545284	614431	301954	545284	614431	301954
	VCAM-1	0	0%	0	0%	0	0%	3300000	3822748	2774715	3300000	3822748	2774715
IV	MMP-1	3	1%	0	0%	3	1%	1508	2654	4021	1517	3029	5515
	MMP-8	0	0%	0	0%	0	0%	21420	38068	55423	21420	38068	55423
	MMP-9	19	6%	0	0%	19	6%	10661	22758	38480	12209	46289	99356
V	C5/C5a	0	0%	0	0%	0	0%	20311	27081	21269	20311	27081	21269
	Collagen-IVα1	0	0%	0	0%	0	0%	1744	2317	2058	1744	2317	2058
	Fas-Ligand	0	0%	0	0%	0	0%	25	34	31	25	34	31
	Galectin-3	0	0%	0	0%	0	0%	3096	3373	1366	3096	3373	1366
	G-CSF	16	5%	0	0%	16	5%	422	7263	33274	454	28764	97703
	I-alpha-1/COL1A1	29	9%	0	0%	29	9%	7552	8900	6515	7926	22519	42846
	MPO	1	0%	0	0%	1	0%	34596	39053	22726	34762	39424	23605
	Pentraxin-3	0	0%	2	1%	2	1%	7007	13606	19296	6959	13518	19264
	Resistin	0	0%	0	0%	0	0%	45965	44708	23887	45965	44708	23887
	S100A8	0	0%	0	0%	0	0%	633	1083	2339	633	1083	2339
	S100A9	7	2%	5	2%	12	4%	2197	5455	14986	2213	9707	32129

Thrombomodulin	0	0%	0	0%	0	0%	10228	11332	5970	10228	11332	5970
TNFα	0	0%	0	0%	0	0%	23	43	150	23	43	150

Categories: I-Chemokines, II-Interleukins, III-Soluble adhesion molecules, IV- Matrix metalloproteases, and V-Others. OOR: Out of range, SD: Standard deviation
